# Microbial Blue Bioprospecting: Exploring the Advances of Compounds Post-Discovery

**DOI:** 10.3390/md23100406

**Published:** 2025-10-17

**Authors:** Cristiana Roberta Multisanti, Valeria Celi, Aurora Dibra, Angela Pintus, Rosario Calogero, Carmen Rizzo, Caterina Faggio

**Affiliations:** 1 Department of Veterinary Sciences, University of Messina, 98168 Messina, Italy; cristiana.multisanti@studenti.unime.it; 2 Department of Chemical Biological, Pharmaceutical and Environmental Sciences, University of Messina, Viale Ferdinando Stagno d’Alcontres 31, 98166 Messina, Italy; valeria.celi@studenti.unime.it (V.C.); cfaggio@unime.it (C.F.); 3 Stazione Zoologica Anton Dohrn, Department of Ecosustainable Marine Biotechnology, Sicily Marine Centre, C. da Porticatello, 29-Villa Pace, 98167 Messina, Italy; rosario.calogero@szn.it; 4 Department of Biology and Chemistry, Faculty of Natural Sciences, University “Luigj Gurakuqi” of Shkodra, 4000 Shkodra, Albania; aurora.dibra@unishk.edu.al; 5 National Research Council of Italy (CNR), Institute for Organic Synthesis and Photoreactivity (ISOF), Via Piero Gobetti 101, 40129 Bologna, Italy; angelapintus@cnr.it; 6 Stazione Zoologica Anton Dohrn–CRIMAC, Department of Integrative Marine Ecology, Calabria Marine Centre, C. da Torre Spaccata, 87071 Amendolara, Italy; 7 Institute of Polar Sciences, National Research Council (CNR-ISP), Spianata S. Raineri 86, 98122 Messina, Italy; 8 Stazione Zoologica Anton Dohrn, Department of Marine Biotechnology, Villa Comunale, 80121 Naples, Italy

**Keywords:** bioprospecting, marine bacteria, biotechnology, toxicity, model organisms

## Abstract

Marine biotechnology is an emerging field of research. There is scientific evidence of the strong potential of a multitude of marine microorganisms in biotechnology, with applications spanning the medical, pharmaceutical, cosmeceutical, nutraceutical and environmental recovery fields. However, despite the discovery of new natural compounds being of wide-ranging benefit, their practical application still remains difficult due to costs and lengthy validation processes. The strength of natural compounds is that, unlike synthetic or already-known compounds, they can have more specific functions and are generally environmentally friendly. This requires, however, that each newly discovered compound be assayed for its toxicity through tests on model cells and organisms. Research should therefore not stop with the simple discovery of new compounds but go beyond with the validation of their efficacy and safety, an issue that remains poorly addressed for products of marine bacterial origin. This review analyses current knowledge on natural compounds of marine bacterial origin, trying to focus on the necessary steps after their discovery, including the investigation of their non-toxicity to model organisms.

## 1. Introduction

Natural compounds of biotechnological interest are chemical substances produced by a wide variety of living organisms, including plants, animals, and microorganisms [1,2,3,4,5]. In this context, bacteria synthesize a diverse array of bioactive compounds, known as bacterial metabolites, which serve multiple biological functions and exhibit a broad spectrum of properties and applications. Among these metabolites, antibiotics, enzymes, exopolysaccharides, antibiofilm and antifouling compounds, and biosurfactants have been identified and extensively studied. The ongoing discovery and investigation of such compounds represent a dynamic area of research with significant applications across various fields, including pharmaceuticals, medicine, industry, aquaculture, agriculture, and food production [6].

Interest in compounds of biotechnological concern has surged for several key reasons. First, they are often perceived as safer alternatives to synthetic chemicals, being often non-toxic and generally well-tolerated by other organisms. Second, there is an increasing focus on sustainability, as natural compounds are derived from renewable sources, helping to reduce reliance on finite resources. Additionally, the use of these compounds can minimize the need for costly extraction processes that contribute to environmental pollution. The combination of safety, sustainability, and eco-friendliness has amplified attention on compounds across multiple fields [7,8,9].

A considerable number of compounds have the potential to be developed into therapeutic agents. For instance, several medicinal plants are already known to be sources of compounds that have beneficial effects on human health by acting as anti-microbial infection, anti-cancer, anti-inflammation, and antiviral agents. Some compounds have been demonstrated to exhibit antiviral properties against a range of viral strains, including those belonging to the coronavirus, herpes simplex, influenza, human immunodeficiency, hepatitis B and C virus, and SARS (Severe Acute Respiratory Syndrome) and MERS (Middle East Respiratory Syndrome) families [10]. On the other hand, molecules such as biosurfactants are regarded as a potentially valuable resource for the remediation of environmental contamination, including that caused by hydrocarbon or other apolar pollutants [11].

Although using natural compounds instead of synthetic ones is a proven, sustainable, and safe alternative, these compounds should still undergo rigorous evaluation before they can be marketed and produced on an industrial scale [12]. For several decades, researchers have been investigating new potential sources, and the marine environment has emerged as strongly promising due to its high biodiversity, which provides numerous opportunities for the discovery of new producers and/or products. However, most bioprospecting research has focused on finding natural compounds produced by marine organisms, mainly marine plants and invertebrates, while only recently researchers have started to focus on marine microorganisms [13]. Bacteria, particularly those from marine environments, have gained significant attention among producers of compounds of biotechnological interest, due to their remarkable metabolic adaptability and chemical diversity, their ability to thrive under extreme environmental pressures, which enables them to produce structurally unique and functionally diverse bioactive molecules. Marine bacteria have been found to synthesize a wide range of secondary metabolites, including antibiotics, enzymes, biosurfactants, exopolysaccharides, and antifouling agents. Many of these compounds exhibit promising pharmacological and biotechnological properties [14,15]. These metabolites often arise as ecological responses to the extreme and competitive conditions of marine habitats, providing adaptive advantages such as chemical defense, interspecies communication, and substrate colonization [16].

Unfortunately, with a strong emphasis on compounds of marine bacterial origin, many studies are confined to the discovery phase of the compound, leaving significant gaps in our knowledge of the validation and approval processes. The large number of studies reporting the discovery of new marine bacterial compounds confirm the enormous potential of microorganisms as source of newer and more accessible marine natural products in the field of bioprospecting [17]. The applications are varied and cover all areas of biotechnology, including the medical, pharmaceutical, nutraceutical, and environmental fields. The general impression is that all studies stop at the discovery or optimization phase of the compound, without progressing any further. However, entering compounds into the validation process is essential for their practical use, adding value to the efforts made to discover them. This is true in both the clinical and environmental fields. In the clinical field, it is essential to demonstrate the validity and safety of compounds to be introduced to the market. In the environmental field, it is necessary to ensure that no damage is caused to the environment or its organisms [18]. Introducing compounds into specific trials is certainly a complex and expensive task. For this reason, it may be useful to incorporate specific tests into common biodiscovery workflows that can provide information on the toxicity levels and potential effects of such compounds in model systems. Continuously introducing these assays into screening pipelines could therefore provide additional information and facilitate the introduction of compounds into the approval and marketing process.

Based on this, the aim of this review is to stimulate thought on the biodiscovery of molecules produced by marine bacteria. It also highlights the urgent need to define specific, standardized protocols to ascertain the non-toxicity of compounds, thereby ensuring safer application in the field of bioremediation. The review provides also an overview of natural molecules produced by marine bacteria and explores the main potential biotechnological applications.

## 2. Materials and Methods

For the present study, scientific articles and studies were selected based on their relevance, originality, and scientific quality. The literature search was conducted using databases such as Google Scholar, PubMed, Scopus, and Web of Science, with a focus on articles published from starting from January 2016 up to the most recent publications of the current year (2025). It was necessary to mention some earlier papers to establish a more solid background. Keywords used in the search included combinations of “marine bacteria”, “bioactive compounds”, “secondary metabolites”, “antibacterial”, “biosurfactants”, “extracellular polymeric substances (EPS)”, “marine enzymes”, “aquatic organisms”, “antifouling”, “aquaculture”, “marine natural products”, and “model organisms”. Considering the huge number of studies and reviews on natural molecules from marine bacteria, in the following sections, we provide an overview for the main classes of bioactive molecules to better introduce the topic. Preference was given to studies relating to the screening of bioactive bacterial molecules on model organisms and their introduction into specific trials post-discovery.

## 3. Marine Microbes as a Source of Bioactive Molecules

The marine environment, which covers over 70% of the Earth’s surface, has historically been a rich source of compounds, many of which hold promise as drug candidates [19,20]. Recent decades have seen a growing body of evidence indicating that the marine ecosystem, due to its distinctive array of habitats and biodiversity, represents a vast reservoir of biologically active natural chemicals produced by marine organisms across a range of taxonomic groups [21,22,23].

Marine microbes have a remarkable ability to rapidly sense and adapt to their environment, often producing unique secondary metabolites as a means of defense and survival. From an ecological perspective, many marine microbial natural products act as chemical defenses, evolving into highly potent inhibitors of physiological processes in the prey, predators, or competitors of the organisms that produce them [24].

Marine bacteria have attracted particular interest in recent years due to their ability to grow in laboratory conditions, the potential to optimize biosynthetic processes and their rapid growth rate, which allows for more concrete applications. Unlike many synthetic compounds, these bacterial-derived metabolites are frequently biodegradable, exhibit low toxicity to non-target organisms, and align well with sustainability principles [16,25]. Moreover, using microorganisms as a source of products with biotechnological relevance also has the advantage of solving problems related to extensive sampling and the significant impact on higher organisms. It also guarantees sufficient yields for late pre-clinical and clinical development [26]. As a result, marine bacteria are increasingly recognized as optimal sources of novel natural products, and also ideal candidates for developing truly sustainable, effective and marine ecosystem-friendly solutions in the medical, industrial, and environmental fields.

These compounds are produced during normal metabolic processes or in response to environmental stress and have shown significant potential in both biotechnological applications. Due to their distinctive characteristics, bioactive molecules derived from bacterial marine sources have emerged as a pivotal area of focus within modern biotechnology [27]. They exhibit considerable variability in terms of both their chemical composition and the biological activities they exert. This is due to the variety of living conditions to which marine microorganisms must adapt, which involves modulating their secondary metabolism and developing suitable strategies. However, the current body of knowledge is still limited, and several aspects require further investigation.

The full potential of marine microbial biodiversity and the range of bioactive metabolites they produce remains poorly known. Indeed, few molecules have been extracted from marine microorganisms because many environments are still unexplored. As an example, extreme environments, which are difficult to access and inhospitable to humans, are scarcely explored in the biotechnological field. These environments are characterized by extreme conditions of temperature, salinity, osmolarity, pH, or pressure. Extremophilic bacteria thrive in these environments by developing special survival strategies, including the production of various biomolecules that support their growth and reproduction. Extremophiles possess remarkable adaptations like tolerance to extreme heat, cold, salinity, and pressure [27]. For this reason, remote areas such as polar regions, hypersaline waters, and deep-sea environments hold great potential for discovering unique bioactive compounds of microbial origin with biotechnological and medical applications [28]. For example, psychrophilic enzymes are produced by microorganisms adapted to cold environments, while other bacteria produce heat-resistant enzymes that remain stable at high temperatures [29].

Bacteria use metabolites as chemical signals to interact with their environment and other species [30]. By 2016, over 1277 new compounds with interesting biological functions had been discovered in marine organisms, many of which were from bacteria [31]. These metabolites are classified based on their roles, how they are produced, and are also grouped into primary and secondary metabolites. Primary metabolites provide energy for basic functions such as growth and development, while secondary metabolites are more complex compounds with diverse physiological roles. Recent research shows that the active phase of synthesis for these molecules occurs at the end of the exponential phase and the beginning of the stationary phase of bacterial growth [13].

Researchers are increasingly turning to the study of bacterial genomes, which encode the enzymes responsible for biosynthetic pathways that produce primary metabolites and transform small protein molecules into secondary metabolites. These specialized metabolites regulate cell growth, signal transduction, nutrient acquisition, and interspecies communication and competition, making them potential alternatives to traditional antibiotics [32]. It is estimated that approximately 70% of secondary metabolites identified in marine bacteria are composed of non-ribosomal peptides (NRPs) or hybrid families of non-ribosomal peptide–polyketides with diverse biological activities [33,34,35]. Given this growing scientific interest, the following sections will present information on selected bacterial metabolites of marine origin, highlighting their biological activity and biotechnological potential.

### 3.1. Drugs and Antibiotics

As extensively reviewed by Haque et al. [36], most of the approved marine drugs are used for the treatment of various cancers and are derived from marine animals, i.e., sponges, tunicates, mollusks, and fish. Although it has been hypothesized that symbiotic bacteria may be responsible for producing these metabolites, in many cases, this has not been ascertained [13].

In recent times, the urgent need for new antibiotics has driven the exploration of uncharted marine environments in search of novel bioactive natural products with inhibitory activities. The widespread synthesis of numerous antibacterial compounds has led to excessive use, triggering the phenomenon of antibiotic resistance, which first emerged in the 1980s and has now become a global issue. According to experts from the World Health Organization, by the mid-21st century, antibiotic resistance is expected to reach a level that poses a serious global health threat [19].

Marine microbiota is considered a crucial resource for the discovery and development of new antibiotics. Bacteria synthesize metabolites with inhibitory or bactericidal effects as survival mechanisms, offering protection from external threats and serving as a defense strategy. The discovery and application of antibacterial compounds have dramatically reduced the number of epidemics and fatalities, consequently increasing human life expectancy. For example, life expectancy in Russia was around 30 years in the late 19th century, but by 1962 it had risen to approximately 73 years [37]. Despite significant advancements in medicine, diagnostics, and the treatment of infectious diseases, pathogenic microorganisms continue to pose a serious threat to global human health. This impact is especially severe in developing countries, where access to medications is limited, and in developed nations, where the uncontrolled use of antibiotics has led to the widespread emergence of antibiotic-resistant bacteria [38]. Efforts to create new synthetic antibiotics by modifying existing natural ones have proven largely ineffective, as pathogens often develop resistance after initial testing. The World Health Organization (WHO) has expressed legitimate concerns about the future of humanity in this regard, urging the search for new antimicrobial agents that could serve as alternatives to current antibiotics [32]. One of the most promising strategies for discovering new antibiotics involves exploring the metabolic products of marine bacteria, which have been proven to produce various molecules with antibiotic properties [19,38].

Several promising antibacterial compounds have been identified from marine bacteria, many of which show potent activity against multi-resistant strains and pathogens of great concern for animal and humans. For instance, members of the phylum *Actinobacteria* include several Gram-positive bacterial strains known for their antibiotic production. They are mainly affiliated with the *Actinomycetaceae* family and genera such as *Actinobaculum* and *Streptomyces* [39], which are reported as producers of compounds with unique chemical structures that are toxic to other microorganisms. For instance, anthracimycin, which is a polyketide antibiotic isolated from a marine *Streptomyces* sp. [40], has demonstrated strong inhibitory effects against *Bacillus anthracis*, the causative agent of anthrax [36]. Its unique structure and mechanism of action make it a candidate for dealing with resistant Gram-positive bacterial infections.

Similarly, Streptoseomycin, which is isolated from cultures of the marine-derived *Streptomyces seoulensis*, exhibits a broad spectrum of antibacterial activity. It has been reported to inhibit the growth of both pathogenic and commensal bacteria, including *Helicobacter pylori*, *Staphylococcus aureus*, *Micrococcus luteus*, *Lactobacillus acidophilus*, and *Bifidobacterium bifidum* [41], highlighting its potential for pharmaceutical and probiotic applications.

Broxichinoline, derived from marine *Streptomyces* sp., has also shown significant antibacterial effects [42], although its precise mechanism is still under investigation. It appears to act by disrupting the integrity of the bacterial membrane, making it a promising agent for topical or surface antimicrobial formulations. Furthermore, Ambiguine K and M isonitriles, secondary metabolites of the marine cyanobacterium *Fischerella ambigua*, show potent antibacterial activity against *Mycobacterium tuberculosis* [43]. These compounds, with their unique isonitrile functional groups, represent a little-explored class of antibacterial agents of great relevance for drug development against tuberculosis. Other notable example is Dentigerumycin E, a cyclic peptide produced in co-culture by *Streptomyces* sp. and *Bacillus* sp., which shows not only antibacterial properties but also antiproliferative effects against cancer cells [44]. These compounds, with their different modes of action, underline how the marine environment is a largely unexploited reservoir of antibacterial agents. Their discovery not only expands the antibiotic arsenal, but also offers new molecular scaffolds for the development of next-generation antimicrobials.

Despite their enormous biotechnological potential, actinobacteria are not the only bacteria capable of synthesizing interesting antibiotic compounds. Marine bacteria from polar environments belonging to other taxonomic groups, i.e., Gammaproteobacteria, Firmicutes, and Cyanobacteria, have been extensively reported [45,46,47,48,49,50,51,52] and reviewed by Rizzo and Lo Giudice [13]. Not only antibacterial but also antifungal, antiparasitic, antiproliferative, and antimycotic activities were described for compounds obtained by many bacterial affiliates, namely, *Janthinobacterium*, *Flavobacterium*, *Bacillus*, *Rummeliibacillus*, *Paenibacillus*, *Sporosarcina*, *Pedobacter*, *Arthrobacter*, *Psychrobacter*, and *Rhodococcus* ([13] and the references therein). Recently, sponge-associated bacteria were proven to be producers of volatile bioactive compounds (VOCs) with activity against *Burkholderia cepacia* complex (Bcc) strains [53].

However, these are just a few examples of the multitude of compounds with inhibitory activity isolated from marine bacteria, despite none of them having entered a validation phase or being included in a clinical trial, to the best of our knowledge. In a recent review, Mohan et al. [54] report a detailed list of bacterial compounds clinically used as anticancer drugs, or with other medical and pharmaceutical applications, some of which can be found as patents or inventions registered in public databases, i.e., PubChem, Espacenet or Google Patent. These include peptide compounds (gleomycin, epoximicin, romidespin, antimycin, romidepsin), antibiotics (mitomycin C), nucleosides (pentostatin), lactones (epothilones, rapamycin), and glycosides (doxorubicin, chartreusin). Among them, only some exceptions, namely, Salinosporamide A, Thiocoraline and N-(2-hydroxyphenyl)-2-phenazinamine are produced by a marine bacterium (described below). Indeed, the bacterial producers are mainly bacteria affiliated with different species of *Streptomyces*, *Actinomyces*, and *Actinomadura*, typically isolated from soil or rocks, and therefore of terrestrial origin.

The compounds produced by marine bacteria and already included in clinical trials or patented are few and mostly investigated for the treatment of human diseases. To the best of our knowledge, as also recently reported in Marine Drugs by Haque et al. [36], only Marizomib (deriving from a marine bacterium) is being investigated in clinical trials. Based on the latest research and recent reviews, there are currently no other marine-bacteria-derived antibacterial drugs in clinical trials. There are marine-derived antibacterial candidates that remain in the preclinical phase.

**Figure 1:**
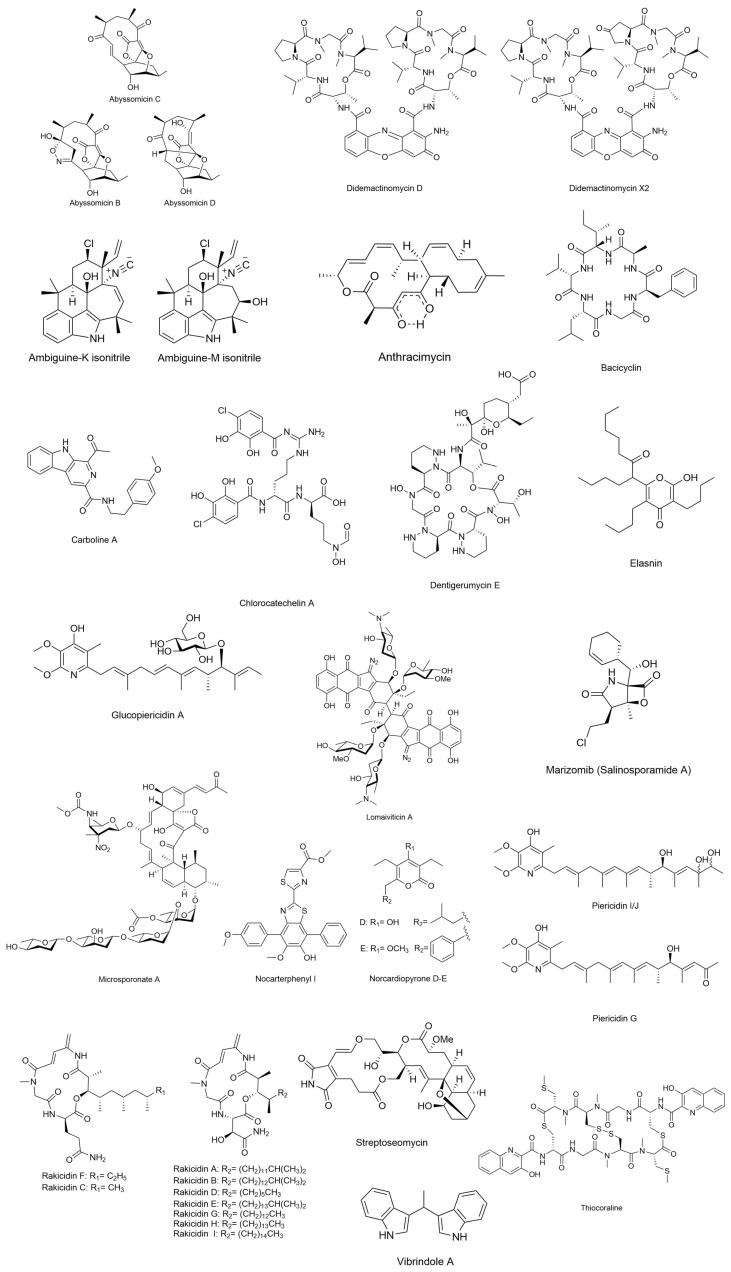
Chemical structure of main drugs or antibiotics of marine bacterial origin under patent application or in clinical trial stage. Figure drawn with ChemDraw 20.1.1.

In the following text, we report some examples of compounds of marine bacterial origin previously reported and deposited [36,54,55]. Table 1 reports examples of the most representative drugs or antibiotics produced by marine bacteria, while Figure 1 shows the molecular structure of main compounds. The only exceptions to this are *Didemactinomycin D* and *Glucopiericidin A*, not produced by marine bacteria but included for their scientific importance. The patents reported were retrieved from freely available databases such as Espacenet, Clarivate Analytics, Patbase, Questel Orbit, Google Patent, and Chemical Abstracts as suggested and reported by Banerjee et al. [55].

#### 3.1.1. Abyssomicins

Abyssomicin is a new class of polyketide antibiotics; abyssomicins were discovered in 2004 and are produced by the actinomycete *Verrucosispora* strain AB 18-032 in the deep-sea sediment of Japan at a depth of 289 m [56]. The isoforms B-D of abyssomicins were obtained after bioassay-guided isolation and identified by NMR and single-crystal X-ray crystallographic analysis. Further chemical isoforms (G, H, atrop-abyssomicin) were then obtained with subsequent studies [57]. Antibiotic and antituberculosis activities were demonstrated for Abyssomicin C [56] and atropabyssomicin C [57]. Controversial observations have been reported to date regarding the central role of enone functionality for the activity of abyssomicin-class compounds [56,57,58]. The p-aminobenzoic acid biosynthesis pathway, which is targeted by abyssomicins, is present in microorganisms but absent in humans. This makes it a promising candidate for the next generation of antibiotics [57]. Other abyssomicins were isolated from other marine *Streptomyces* strains and reported for antiretroviral activity, thus being suggested as ideal candidates for the cotreatment of HIV [59].

#### 3.1.2. Carboline A

Carbolines are alkaloids presenting several chemical isoforms with promising biological activities. Carboline A was obtained from the strain *Actinoalloteichus* sp. ZZ1866 isolated from a sea mud sample in China [60]. In total, 17 alkaloids were isolated, including marinacarbolines E–Q, caerulomycin N, and actinoallonaphthyridine A. Marinacarbolines E–Q are β-carboline alkaloids. The *Actinoalloteichus* sp. ZZ1866 extracts showed antiproliferative activity against human glioma cells.

#### 3.1.3. Didemactinomycin D

Actinomycin D is a pigment peptide lactone antibiotic, firstly isolated from Actinomycetes by Waskman and Wooddruff in 1940 [61]. The strain was further investigated for antibacterial activities later [62].

It is composed of a chromophore (actinocin, 2-amino-4,6-dimethylphenoxazine-3-one-1,9-dicarbo xylic acid) and two cyclic pentapeptones (α ring and β ring) connected to it by an amide bond and appears as a pronounced orange or red compound.

This product can penetrate the cell membrane and selectively aggregate nucleic acids through a binding between the nucleus of phenoxazinone chromophore and the guanine of DNA. Actinomycin has significant antibacterial, antitumor, and antiviral biological activities. It acts by penetrating most cell membranes through osmosis and selectively aggregates in the nucleus, with DNA as the target. The cyclopentalactone can be inserted into the small groove of the DNA to increase the stability of the binding, thereby inhibiting the DNA. Antibacterial, antitumor, and antiviral effects were proven for this compound. It can inhibit tumor cells by affecting nucleic acid synthesis, inducing cell differentiation and apoptosis, affecting the cell cycle, and inhibiting protease activity. Moreover, Actinomycin D can specifically down-regulate the expression of proteins promoting the failure of stem cell populations, preventing breast cancer cells from increasing in value. Low-dose actinomycin D can also specifically activate p53 gene-dependent transcription to accelerate human tumor cell apoptosis. Although demonstrating excellent properties in the treatment of several diseases (chorionic disease, melanoma, renal blastoma, malignant endothelial cell myelopathy, soft tissue sarcoma), toxicity to the human body was also observed in terms of leukopenia, thrombocytopenia, and anemia manifestations. Under the reference of Patent number CN110669103A (https://patents.google.com/patent/CN110669103A/en?oq=CN110669103A accessed on 16 October 2025), the invention of a didemethylated actinomycin derivative for the treatment of drug-resistant bacterial infections has been reported. The compound has been obtained from a methyltransferase-inactivating mutant, *Streptomyces costaricanus* SCSIO ZS0073 [62].

The actinomycin demethylation derivative Didemactinomycin D exhibited good inhibitory activity (MIC is 4–32 μg/mL) on 14 strains of *Staphylococcus aureus* and lower toxicity to normal human cells than actinomycin D [63].

#### 3.1.4. Glucopiericidin A

Glucopiericidin A is deposited as a patent (CN109384823, https://patents.google.com/patent/CN109384823A/en?oq=CN109384823 accessed on 16 October 2025) and is obtained by fermentation, extraction and purification from *Streptomyces* sp. HBERC-58855 cultures. Two phachomycin glucosides were obtained, namely, glucopiericidin A and 7-demethylglucopiericidin A, and proposed as lead compounds for the development of anti-renal cancer drugs.

The patent invention is mainly related to piericidin A, which mainly increases the chemical structure of the glucosyl group, which not only enhances the anti-renal cancer activity, but also increases the polarity of the compound [64].

In the patent application, it is reported that Glucopiericidin A has inhibitory activity on human renal cell carcinoma ACHN cells, while 7-Demethylglucopiericidin A has strong broad-spectrum inhibitory activity on three kidney cancer cells ACHN, OS-RC-2 and 786-O. It describes the use of the two phenomycin glucosides for the preparation of an anti-kidney cancer drug.

#### 3.1.5. Lomaiviticin A

Lomaiviticin A is a dimeric diazofluorene produced by the marine bacterium *Salinispora pacifica* DPJ-0019 [65,66,67]. Despite the chemical similarity with kinamycins, this natural product family exhibits additional, unique structural features of particular interest to chemists. The lomaiviticins are C2-symmetric diazofluorene dimers, with the two halves of the molecule connected via a sterically congested C–C bond. They are also glycosidated with the unusual sugars N,N-dimethyl-L-pyrrolosamine and L-oleandrose. Finally, the lomaiviticins are more extensively oxidized than the kinamycins, possessing an additional A-ring hydroxyl group and a D-ring carbon at the ketone oxidation state. These peculiarities in the chemical structure intrigue chemists and prompt researchers to investigate their mechanism of action and production pathways.

The two classes of natural product diazofluorene exhibit potent cytotoxicity against human cancer cell lines, with lomaiviticin A displaying the greatest activity. The A and B isoforms induce double-strand DNA breaks through mechanisms involving vinyl radical intermediates. They leverage their dual diazofluorene moieties to coordinate efficient DNA backbone scission [68]. Lomaiviticins C–E lack this robust DNA-damaging capability and exhibit comparatively lower cytotoxicity. The rarity of lomaiviticins in nature, coupled with the challenges of their total synthesis, offers unique opportunities for the discovery of new analogs through both natural product exploration and innovative synthetic methodologies.

#### 3.1.6. Marizomib (Salinosporamide A)

Salinosporamide A NPI-0052, also known as Marizomib, is a proteasome inhibitor with γ-lactam-β-lactone bicycle core structure, derived from by the obligate marine bacteria *Salinispora tropica* and *Salinispora arenico* isolated from ocean sediment [66]. The compound can permanently block three proteasome activities: caspase-like (C-L, β1), trypsin-like (T-L, β2), and chymotrypsin-like (CT-L, β5) [69] with great effectiveness ascertained in preclinical studies [70]. It was developed by Nereus Pharmaceuticals for the treatment of multiple myeloma and entered Phase I clinical trials only three years after its discovery in 2003. In addition to its anticancer effect, the compound can cross the blood–brain barrier expanding the range of its therapeutic possibilities. It has been recently demonstrated that it promotes Apoptosis in malignant melanoma cells, specifically A375 and G361 Melanoma Cancer Cells [70].

In phase-I clinical trials, it has been demonstrated that salinosporamide was well-tolerated in relapsed or relapsed and refractory multiple myeloma patients [71]. The compound is now in Phase III Clinical Trials [72].

#### 3.1.7. Piericidin

Piericidins are microbial-derived α-pyridone antibiotics, of which different isoforms have been isolated from actinomycetes of terrestrial and marine origin. The pteridomycins have been reported to have insecticidal and antibacterial activities, and have inhibitory activities on some tumor cells, but the mechanism of action is very weak, and the anti-tumor potential needs further exploration. The invention deposited as CN109384710 (https://patents.google.com/patent/CN109384710A/en?oq=CN109384710, accessed on 16 October 2025) reported three kinds of phenomycin (Piericidin G, Piericidin I and Piericidin J) obtained by fermentation and extraction purification on the mangrove-derived *Streptomyces* sp. HBERC-58855 (patent application number 201710344174.3) [64,73]. The chemical structures of piericidins G, I, and J possess more carbonyl or hydroxyl groups than piericidin A.

The cytotoxicity effects of the phenomycin-like compounds piericidins G, I and J on normal human renal tubular epithelial cells were weak, but have significant inhibitory activities against two kinds of renal cancer cells (OS-RC-2 cells and ACHN cells), selectively. The applicants suggested the use of phenomycin in preparing anti-kidney cancer drugs.

#### 3.1.8. Polypeptide (PBN3)

A new polypeptide with a molecular weight of 759.44 Da and amino acid sequence of Leu-Thr-Ala-His-Tyr-Arg produced by the marine strain *Brevibacillus* sp. N189 is deposited as a patent under the identification number CN113150071 (https://patents.google.com/patent/CN113150071A/en?oq=CN113150071 accessed on 16 October 2025). The producer bacterial strain was isolated from Antarctic seawater and is a Gram-positive and aerobic bacterium, with salt tolerance up to 6.5% NaCl (*w*/*v*), an optimal pH range of 5.5–8.5, and a temperature range of (12–37 °C) [74].

The compound was applied for the preparation of drugs useful in the treatment of several diseases, i.e., lung cancer, liver cancer, pancreatic cancer, breast cancer or cervical cancer. The invention provided the base for the development of an anti-tumor drug effective against human non-small-cell lung cancer cells A549, human large cell lung cancer cells NCI-H460, human liver cancer cells HepG2, human pancreatic cancer cells Panc 28, breast cancer MCF-7 and human cervical cancer cells.

#### 3.1.9. Rakicidin

Rakicidin compounds are important compounds known for their antitumor or antibacterial activity. Several isoforms have been obtained from *Streptomyces* spp. and *Micromonospora* spp. [75,76,77,78]. Recently, the cytotoxic activity of Rakicidin A, B and B1 against five human tumor cell lines, HCT-8, MGC803, A549, A375, Hep G2 and CASKI, was determined, as well as the apoptosis induction of myeloid chronic leukemia stem cells [75].

The patents deposited under n° CN108329280 (https://patents.google.com/patent/CN108329280A/en?oq=CN108329280 accessed on 16 October 2025), CN108586380 (https://patents.google.com/patent/CN108586380A/en?oq=CN108586380 accessed on 16 October 2025), and CN110698537 (https://patents.google.com/patent/CN110698537A/en?oq=CN110698537 accessed on 16 October 2025) report the compounds Rakicidin I and Rakicidin H from *Micromonospora* sp. FIM 02-523 and Rakicidin B1-2 from *Micromonospora* sp. FIM-R160609 with excellent hypoxic selective anti-tumor cell activity and anti-colon cancer HCT-8 cell activity. Specifically, the inventors proposed a method of extraction to provide natural Rakicidin-like compounds Rakicidin I and Rakicidin H [79,80,81].

#### 3.1.10. Thiocoraline

Thiocoraline is a depsipeptide with anticancer activity isolated from the marine strain *Micromonospora marina*. It influences and interacts with the cell cycle by blocking the G1 phase and the decline of the S phase in human colon cancer cell lines. It also exhibits antimicrobial activity against Gram-positive microorganisms, i.e., *Staphylococcus aureus*, *Bacillus subtilis* and *Micrococcus luteus* [82], and inhibits RNA synthesis.

It has been retrieved from a thiocoraline analogue, 22′-deoxythiocoraline, isolated from the sponge symbiont *Verrucosispora* sp. WMMA107 with strong cytotoxic activity against human alveolar epithelial A549 cells [83]. The activity of thiocoraline against the human medullary thyroid carcinoma MTC-TT cell line was shown to be produced by cell cycle arrest in the G1 phase and activation of the Notch signaling pathway [84]. To the best of our knowledge, Thiocoraline is under preclinical pharmacodynamic evaluation by PharmaMar and is a promising compound for approval to enter Phase I clinical trials [85]. 

**Table 1:** List of the most representative compounds of drugs or molecules with biological activity produced by marine microorganisms, with details of activity, patent number of clinical trial stage, if applicable.

Compound	Origin	Biological Activity	Type and Chemical Structure	Patent n°	References
**Anthracimycin**	*Streptomyces* sp.	Inhibitory effect against some pathogenic bacteria such as *Bacillus anthracis*, whose spores cause anthrax.	Macrolide polyketide; C_25_H_32_O_4_	N/A	[86]
**Nocarterphenyl I/Nocardiopyrone D**	*Nocardiopsis* sp.	Antibacterial activity against *B. subtilis* and *E. coli*	p-terphenyl and α-pyrone	N/A	[63]
**Vibrindole A**	*Vibrio parahaemolyticus*	Antimicrobial activity against *S. aureus*, *S. albus* and *B. subtilis*	N,N-Diphenyl-p-phenylenediamine C_18_H_16_N_2_	N/A	[87]
**Dentigerumycin E**	*Streptomyces* sp. and *Bacillus* sp.	Antiproliferative and antimetastatic activities against human carcinoma	Cyclic depsipeptide; C_40_H_67_N_9O_	N/A	[44]
**Bacicyclin**	*Bacillus* sp.	Antibacterial activity against *E. faecalis* and *S. aureus*	Cyclic hexapeptide; C_31_H_48_N_6_O_6_	N/A	[88]
**Chlorocatechelin A**	*Streptomyces* sp.	Inhibited the growth of a wide range of bacterial and fungal pathogens	Acylguanidine-type siderophore; C_26_H_30_Cl_2_N_6_O_11_	N/A	[89]
**Ambiguine-K and M isonitrile**	*Fischerella ambigua*	Antibacterial activity against *Mycobacterium tuberculosis*	Indole alkaloid with isonitrile group; C_26_H_29_ClN_2_O and C_2_H_3_N	N/A	[15]
**Streptoseomycin**	*Streptomyces seoulensis*	Antibacterial activity against *Helicobacter pylori*, *Lactobacillus acidophilus*, *Bifidobacterium bifidum*, *Eubacterium brachy*, *Propionibacterium acnes*, *Staphylococcus aureus*, *Micrococcus luteus* and *Bacillus subtilis*	Aminoglycoside; C_31_H_37_NO_11_	N/A	[90]
**Microsporonates A**	*Micromonospora harpali*	Antibacterial activity against Gram-positive bacteria	Complex polycyclic macrolides	N/A	[90]
**Patent or Clinical Trials Involved Compounds**					
**Abyssomicin**	*Verrucosispora* strain AB 18-032	Antibiotic, Antiretroviral	Polyketide	CN110092758A	[56]
**Carboline A**	Marine bacteria *Actinoalloteichus* *cyanogriseus* ZZ1866	Anticancer	Alkaloid	CN111747955	[60]
**Didemactinomycin D**	*Streptomyces costaricanus* SCSIO ZS0073	Antibacterial activities against *Staphylococcus aureus*	Peptide lactone antibiotic	CN110669103	[91]
**Glucopiericidin A**	*Streptomyces* sp. HBERC-58855	Renal cancer	α-pyridone	CN109384823	[63]
**Lomaiviticin A**	*Salinispora pacifica* DPJ-0019	Induces DNA double-strand breaks	Dimeric diazofluorenes	JP2705789B2	[65]
**Marizomib (Salinosporamide A)**	*Salinispora tropica*	Breast cancer, multiple myeloma	γ-lactam-β-lactone bicycle core C_15_H_20_ClNO_4_	Clinical Trial stage (Phase III)	[36,72]
**Piericidin**	*Streptomyces* sp. HBERC-58855	Renal carcinoma	α-pyridone	CN109384710A	[64]
**Polypeptide PBN3**	Marine bacteria *Brevibacillus* sp. N189	Lung cancer, Liver cancer, Pancreatic cancer, Breast cancer or Cervical cancer	Polypeptide	CN113150071	[74]
**Rakicidin I**	*Micromonospora* sp. FIM 02-523	Colon, Pancreatic cancer	Cyclic lipopeptide	CN108329280	[79]
**Rakicidin H**	*Micromonospora* sp. FIM 02-523	Colon, Pancreatic cancer	Cyclic lipopeptide	CN108586380	[80]
**Rakicidin B1-2** **Depsipeptide**	Marine bacteria sp. FIMR160609	Colon, Pancreatic cancer	Cyclic lipopeptide	CN110698537	[81]
**Thiocoraline**	*Micromonospora marina*	Colon cancer, Medullary thyroid carcinoma	Depsipeptide	Preclinical pharmacodynamic evaluation by PharmaMar	[82]

N/A: not available.

### 3.2. Extracellular Polymeric Substances

Extracellular polymeric substances (EPS) are high-molecular-weight biopolymers synthesized and secreted by microorganisms into their surrounding environment. They are mainly composed of polysaccharides, both homopolysaccharides (HoPS) and heteropolysaccharides (HePS), together with proteins, lipids and minor inorganic components [51]. EPSs can be broadly classified into soluble microbial products (SMPs), which are released during cell lysis or hydrolysis, and bound EPSs, which are more closely associated with cell surfaces [92].

Functionally, EPSs act as a protective matrix, increasing microbial resistance to environmental stressors, toxins and antimicrobials, while facilitating surface adhesion and biofilm formation through interactions with divalent cations such as Ca^2+^ and Mg^2+^ [91]. In biofilms, EPSs often account for up to 90 per cent of the biomass, forming a hydrated, three-dimensional structure that encapsulates microbial communities.

EPSs generally have a gelatinous, sticky, and highly hydrated consistency that acts as a protective shield for microorganisms [93,94]. EPS-producing bacteria have been identified in various environments, and the composition of their EPS varies significantly, containing proteins, lipids, and other minor components such as uronic acid and inorganic substances, with polysaccharides being the major component. The identification and characterization of these compounds largely depend on the extraction methods used [95].

In addition to their ecological role, EPSs are gaining attention for their biotechnological applications, including emulsifying, antibacterial, anti-dehydrating, and metal chelating properties [95]. For instance, EPSs produced by *Pseudoalteromonas* sp. MER144, isolated from Antarctic waters, and *Winogradskyella* sp. CAL384, have demonstrated effective cryoprotective activity and the ability to chelate heavy metals, making them suitable for applications in polar bioremediation [96,97]. EPS produced by *Colwellia* sp. GW185 and *Shewanella* sp. CAL606, both Antarctic sponge-associated strains, showed useful emulsifying activity for oil dispersion [91].

EPSs produced by *Pseudoalteromonas antarctica* and *Vibrio diabolicus* have also shown potential for dermal regeneration; the latter produces a hexosaccharide that is structurally similar to hyaluronic acid [51]. These examples highlight the wide applicability of EPSs, from eco-toxicological protection to cosmeceuticals.

To date, scientific literature and industry data show that only a limited number of marine EPSs have achieved real commercial use, mainly in the cosmetic and dermocosmetic fields rather than in pharmaceuticals. Indeed, according to Jeewon et al. [98], despite widespread interest in microbial polysaccharides and their great potential for numerous applications, only a few of these polymers have actually been commercialized to date, and even fewer come from marine microorganisms, e.g., the EPS HE800EPS and HYD657.

However, there is evidence of existing patents relating to marine microbial polysaccharides that suggest the potential of other polymers. Some EPS patent applications are also reported below, but as reviewed by Jeewon et al. [98], many other patent applications (i.e., CN116120477A, https://patents.google.com/patent/CN116120477A/en?oq=CN116120477A%2c accessed on 16 October 2025; CN105087450A, https://patents.google.com/patent/CN105087450A/en?oq=CN105087450A accessed on 16 October 2025; LU501700B1, https://patents.google.com/patent/LU501700B1/en?oq=LU501700B1 accessed on 16 October 2025; CN109457001A, https://patents.google.com/patent/CN109457001A/en?oq=CN109457001A accessed on 16 October 2025) refer to methodologies for culture of producers and preparation of polysaccharide.

#### 3.2.1. EPS HE800 (Hyalurift^®^)

HE800 EPS, produced by the *Vibrio diabolicus* bacterium HE800 strain (CNCM number: I-1629), is very peculiar. Indeed, HE800 EPS (Hyalurift^®^ trademark) has a glycosaminoglycan-like structure, constituted by a linear non-sulfated tetrasaccharidic repeating unit, including equal amounts of glucuronic acid and hexosamine (N-acetyl-glucosamine and N-acetyl-galactosamine). It has been described as the combination of both hyaluronan and non-sulfated chondroitin disaccharidic repeating units, therefore combining both high uronic acid and hexosamine contents. Heavy metal chelating activity was proven for HE800, as well as enhancement of in vivo bone repair in a rat model [98]. HE800EPS has great potential for tissue regeneration in vitro, is capable of restoring bone and skin, and can accelerate collagen fibrillation in vitro and activate fibroblasts [99].

#### 3.2.2. EPS HYD657 (Abyssine^®^)

Abyssine is the first example of marine EPS commercially exploited in the cosmetic industry [100]. It is produced by an *Alteromonas macleodii* subsp. *fijiensis* bacterium, with a high molecular weight (1.5 × 10^6^ Da) and a monomer composition as follows: Glc/Gal/Man/Rha/Fuc/GlcA/GalA (molar ratios: 1/1.9/0.4/0.6/0.2/1.2/2.8). It is commercialized for the functions involved in the protection of sensitive skin against chemical, mechanical and UVB aggressions.

#### 3.2.3. EPS WO2015117985A1

The Application for this EPS is patented under the Patent Cooperation Treaty with number WO2015117985A1 (https://patents.google.com/patent/WO2015117985A1/en?oq=WO2015117985A1, accessed on 16 October 2025). The EPS is produced by the cold-adapted bacterium *Pseudomonas* sp. CECT8437, isolated from a marine sediment sample of Deception Island (South Shetland Islands, Antarctica). The EPS is composed of glucose, galactose, fucose and uronic acid in a molar ratio of approximately 2:1:1:0.3, with the following approximate weight percentages: 37.29% C, 6.17% H, 2.25% N, and 0.41% S. Its molecular weight (MW) is higher than 2 × 10^6^ Da. The application suggested the possible use of the EPS in cosmetic compositions, as it exhibited several functions and better performance than other characterized EPSs, i.e., as a cryoprotectant agent; emulsifier agent; thickening, stabilizing or structural agent; dermoprotective agent; and agent for increasing skin elasticity.

#### 3.2.4. EPS AU2016330332B2

The EPS is available under the deposition number AU2016330332B2 (https://patents.google.com/patent/AU2016330332B2/en?oq=AU2016330332B2, accessed on 16 October 2025) and is a low-molecular-weight (15 kDa) over-sulfated exopolysaccharide (GYS15). It is produced by a *Alteromonas* sp. isolated from deep-sea hydrothermal environment. The invention proposed the possible use of EPS for its anti-metastatic and cancer treatment.

#### 3.2.5. EPS ES2585398B1

The EPS isolated is obtained from cultures of *Pseudomonas* sp. CECT8437, a strain isolated from marine sediment. The structure is composed of glucose, galactose, fucose and uronic acid in a molar ratio of approximately 2:1:1:0.3 and possesses an elemental composition of approximately 37.29% C, 6.17% H, 2.25% N and 0.41% S in weight percentages. The invention was aimed at providing cosmetic compositions comprising effective amounts of the EPS and cosmetically acceptable ingredients or carriers. The PES showed cryoprotective, emulsifying, thickening, stabilizing, and texturizing activity. Interestingly, the production of EPS is not accompanied by the release of external membrane vesicles (VMEs) by bacteria, as normally occurs in most *Pseudoalteromonas* strains. This potentially represents an advantage of the invention, as the presence of vesicles can affect the extraction and purification and/or increase the toxicity and/or antigenicity, since it has been shown that the vesicles contain a wide variety virulence factor. According to the applicants, the commercial value of the EPS of the invention is based mainly on this property.

#### 3.2.6. EPS US10993434B2

The EPS was obtained by a new strain inhabiting polar region, namely *Pseudoalteromonas* sp. strain CY01 (KCTC 12867BP). The patent is focused on the cell cryoprotectant activity of the compound, which shows no cytotoxicity. Therefore, the EPS is proposed as an optimal alternative to conventional cryoprotective agents with cytotoxicity when used at high concentrations.

### 3.3. Biosurfactants

Biosurfactants (BS) are amphiphilic compounds produced by marine bacteria that reduce surface and interfacial tension, enabling the emulsification and solubilization of hydrophobic substances such as hydrocarbons, lipids and oils [13]. Structurally, BS contain a hydrophobic part, typically composed of fatty acids or lipids, and a hydrophilic component consisting of peptides, amino acids or carbohydrates [99,101]. Due to their amphiphilic molecular structure, BSs act by reducing surface tension between substances with differing polarities, promoting the production of foams, and improving the emulsification [102]. BSs are generally divided into two classes: low-molecular-weight compounds, such as rhamnolipids, lipopeptides and sophorolipids, which are effective in reducing surface tension, and high-molecular-weight compounds, including lipopolysaccharides, glycoproteins and polymeric emulsifiers, which stabilize emulsions [103]. Among the most interesting molecules of marine microbial origin, BSs are gaining attention in biotechnology due to their potential use in cleaning up contaminated and damaged environments [103]. These natural surfactants also show promise for applications in industries like detergents, food production, chemical manufacturing, and pharmaceuticals. Most of the studies related to BS of marine microbial origin are focused on temperate environments and BS applications in the field of bioremediation. The genera *Pseudomonas*, *Bacillus*, *Rhodococcus*, *Candida* and *Halomonas* are well known in the field of biosurfactant production. For instance, the rhamnolipids of *Pseudomonas aeruginosa* are known for their role in bioremediation and biofilm inhibition and have been studied for antifouling paints due to their ability to modify surface hydrophobicity and prevent microbial adhesion [103]. BS produced by *Bacillus niabensis* showed high emulsifying activity and low toxicity to *Artemia franciscana*, which supports its use in marine antifouling coatings and environmental cleaning. Similarly, emulsan, produced by *Acinetobacter venetianus* RAG-1, has amphiphilic properties and is used commercially in industrial cleaning formulations. Mannosylerythritol lipids from *Pseudozyma* sp. have demonstrated antimicrobial and surfactant properties, being biodegradable and well tolerated by the skin [104]. To date, bacterial BS producers have generally been isolated from abiotic matrices of contaminated environments, but also extreme environments and marine invertebrates have recently been identified as additional potential sources of new BS producers. Gammaproteobacteria isolates affiliated to the genera genus *Vibrio*, *Providencia*, *Psychrobacter*, *Halomonas*, *Pseudoalteromonas*, *Idiomarina*, *Marinobacter* sp., *Alcanivorax* sp. and *Acinetobacter* sp. have been isolated from marine invertebrates. Despite less represented, bacterial genera in Alphaproteobacteria and Betaproteobacteria groups have also been detected with *Pseudovibrio* and *Alcaligenes* sp. affiliates, respectively isolated from different species of marine invertebrates, i.e., the sea pen *Pteroeides spinosum*, the coral *Acropora digitifera*, the sponges *Dendrilla nigra* and *Callyspongia diffusa* (extensively reviewed by Rizzo and Lo Giudice [13]. The use of biological matrices as a source of bacterial biosurfactant (BS) producers has proven to be an effective method of detecting new compounds, and is an emerging, appealing approach.

The vast structural diversity of biosurfactants and the consequent broad range of properties justify why they continue to pique scientific interest, and the occurrence of claimed patents [105,106]. To the best of our knowledge, no marine bacterial biosurfactants have been launched on the market to date. All available evidence indicates that these compounds remain in the pre-commercial research phase, with some promising initiatives (e.g., Marisurf, according to Voulgaridou et al. [107], but no established commercial products. The strain *Acinetobacter venetianus* RAG-1 strain produces the bioemulsant emulsan, widely described in literature and subject to pilot-scale production and several industrial patents. Despite its strong application potential (e.g., industrial cleaning, bioremediation) [107], there is no evidence of its actual commercialization in finished products.

Silva et al. [106] listed the patents on biosurfactants produced by microorganisms, but there is no clear reference to biosurfactants produced by marine microorganisms. Additionally, as with EPS, most applications in this field refer to the bacterial producer or the method used to obtain the biosurfactant. They are mostly sophorolipids, rhamnolipids and lipopeptides, mainly produced by *Pseudomonas* spp., *Candida* spp. or *Streptomyces* spp., but in many case the origin and the phylogenetic affiliation is not specified.

The invention available under the number WO2012164508A1 reports the method for producing a biosurfactant tensioactive extract produced by the strain *Cobetia marina* strain (MM1IDA2H-1), CECT No. 7764, with potential application for the prevention of infectious pathologies in aquaculture, as an additive in paint formulations or in food for fish, which induces an immune response.

In line with this, the main recommendations in the BS production field suggest exploring and ascertain new potential sources for the isolation of bacterial producers, which could be enhanced through the improvement of research on marine symbiotic bacteria and extremophilic bacteria. Moreover, strong efforts should be spent on the optimization processes and on the purification of new molecular forms.

### 3.4. Enzymes

Marine bacteria serve as an abundant source of enzymes with potential applications across various fields, including biotechnology, the food industry, biofuel production, and more. These enzymes, shaped by metabolic evolution in diverse marine environments, possess unique characteristics that make them key molecules for innovative processes, both in terms of methodologies and the resulting products and applications [104].

Enzymes produced by marine microorganisms are often correlated with the adaptations that they develop to thrive in the physical and chemical conditions of their environments. Due to factors such as salinity, temperature, pressure, and other marine-specific environmental conditions, marine enzymes tend to be more stable and capable of functioning in extreme conditions compared to terrestrial enzymes, making them particularly valuable for industrial use. Consequently, marine enzymes have become increasingly prominent in the global industrial enzyme market, with an annual production growth rate of 10–15% [108].

Research on marine enzymes is continuously evolving. Among the enzymes identified so far are bacterial amylases, which break down starches and are useful in producing fermentable sugars from marine sources such as algae; lipases, which catalyze the hydrolysis of lipids and fats; DNases, which hydrolyze DNA into nucleotide fragments; and cellulases, which degrade cellulose found in algae and other plant organisms [109]. In the context of enzymes of marine microbial origin, extremozymes are particularly interesting.

Although numerous natural enzymes with biotechnological and industrial applications have been identified, the current supply remains insufficient to meet growing industrial demand. Researchers have turned their attention to extreme environments as sources of natural enzymes to better address these challenges, in response to the need for enhanced enzyme performance under industrial conditions such as extreme temperatures, pressure, and pH levels. Indeed, extremophilic microorganisms produce extremozymes, namely enzymes capable of functioning under harsh conditions. The use of extremozymes can improve the efficiency of biotechnological processes, lower costs, and expand the potential for utilizing natural resources [110,111,112].

Among these, several thermophilic enzymes (thermo-enzymes), such as cellulases, amylases, proteases, lipases, and others, have been characterized by their unique ability to maintain stable activity at high temperatures. These enzymes possess physical and electrostatic properties that allow them to function efficiently under extreme thermal conditions [113]. In this context, among the most notable extremozymes is Taq polymerase, extracted from the organism *Thermus aquaticus*. This enzyme revolutionized biotechnology by enabling the selective amplification of specific DNA sequences through the Polymerase Chain Reaction (PCR), a fundamental technique now widely used in molecular genetics research.

Similarly, enzymes extracted and purified from barophilic or piezophilic organisms can operate under the high hydrostatic pressures typical of deep-sea environments, which are inhospitable to most life forms. These pressure-adapted enzymes are valuable tools in various biotechnological processes, as they retain their unique catalytic abilities under extreme pressure. Researchers are investigating their operational potential in industrial processes such as biofuel production, pharmaceutical synthesis, and food processing. Additionally, studying these enzymes provides valuable insights into microbial diversity and the adaptations of deep-sea ecosystems [114].

Enzymes produced by acidophilic microorganisms that thrive in pH ranges of 3–4, and halophilic microorganisms [115], which tolerate unique osmotic pressures, are capable of functioning under extreme pH and salinity conditions, respectively. Halophiles are categorized based on their tolerance to NaCl concentrations: slight halophiles (200–500 mM NaCl), moderate halophiles (500–2500 mM NaCl), and extreme halophiles (2500–5200 mM NaCl) [116]. Acidophilic enzymes are often sourced from habitats such as acidic lakes, marine vents, acidic hot springs, and other geological environments with highly acidic conditions. These enzymes are structurally stable due to their unique ionic bonds, disulfide bridges, and tertiary structures, which protect them from denaturation in acidic environments. Their stability makes them ideal for industrial processes requiring acidic conditions, such as fruit juice production, food processing, and chemical manufacturing [117]. They are also studied for their enzymatic mechanisms and kinetics in both basic and applied research [118]. Furthermore, their ecology provides insights into microbial adaptations to acidification, which is particularly relevant for understanding the impacts of ocean acidification due to climate change.

Halophilic enzymes, on the other hand, exhibit greater activity and stability in the presence of NaCl, under both low and high temperatures. These enzymes hold promise for various applications in the food, pharmaceutical, and detergent industries [111]. At present, there is no scientific evidence in the literature regarding registered clinical trials for marine bacterial enzymes. However, several patents are available which, although limited to the preclinical phase, have shown promising results in experimental models, as reported below.

#### 3.4.1. NucB

NucB is a marine-derived nuclease first identified from a marine strain of *Bacillus licheniformis* [119]. Studies have demonstrated its strong ability to disperse bacterial biofilms. Particularly, this enzyme has shown a high capacity to degrade extracellular DNA (eDNA) present in biofilm matrices, leading to the dispersion of bacterial biofilms. Studies have demonstrated its effectiveness against biofilms of bacteria often associated with chronic infections such as chronic sinusitis [120]. In this context, NucB has shown activity against biofilms of *Bacillus licheniformis* strains and other medical bacteria, suggesting potential use in clinical settings such as chronic sinusitis [119]. Today, research on NucB has been limited to the preclinical phase, with experimental evidence derived from in vitro tests and ex vivo models [121,122,123]. Despite the lack of clinical studies, the enzyme has attracted interest as a potential antibiofilm agent for medical applications.

#### 3.4.2. FlAly

Alginate lyase is a marine-derived enzyme whose ability is to degrade alginate, a key component of the extracellular matrix in bacterial biofilms, leading to the dispersal of biofilm-encased cells and increased susceptibility to antimicrobial agents [124]. Nowadays, research on alginate lyase has been limited to the preclinical stage but including a patent protection, sought for its production and application [125,126]. Specifically, a patent is available (CN110195051A, https://patents.google.com/patent/CN110195051A/en accessed on 10 September 2025), that describes optimized fermentation methods using the marine bacteria *Flavobacterium mizutaii* to produce and improve the activity of the enzyme. Moreover, studies have reported clear evidence of the effectiveness of various alginate lyases produced by the marine strain Flavobacterium sp. UMI-01 (FlAlyA, FlAlyB, FlAlyC and FlAlex) belonging to the PL7 family and having the function of endolytically degrading alginate, acting mainly on mannuronate blocks and, in some cases, on guluronate blocks [127]. Despite these developments, no clinical trials have been reported, although alginate lyase remains a promising candidate for antibiofilm strategies in medical and industrial settings.

### 3.5. Anti-Biofilm and Anti-Fouling Compounds

Biofouling is a natural process in aquatic environments, typically occurring in three stages: (1) formation of a primary biofilm of organic materials; (2) colonization by microorganisms like bacteria and diatoms; (3) establishment of larger organisms such as algae and marine invertebrates. Historically, biofouling has been controlled using toxic chemical compounds, including tributyltin (TBT), arsenic, and mercury. These substances were banned due to their non-specific toxicity and environmental persistence.

Current antifouling products often contain copper and zinc, but these also pose ecological risks, especially to non-target marine species. As a result, interest has grown in developing environmentally friendly antifouling alternatives derived from natural sources, particularly from marine organisms. Marine microorganisms, especially bacteria, are promising because they produce a range of bioactive compounds that can prevent the settlement of both microorganisms and macroorganisms. These natural antifouling agents offer a sustainable and less harmful solution for protecting submerged surfaces, such as ship hulls and aquaculture equipment. Moreover, marine bacteria can be cultured under controlled conditions, enabling the large-scale, cost-effective production of these compounds.

At the initial phase of conditioning film formation (from seconds to hours), marine bacterial compounds disrupt the adsorption of organic molecules to surfaces. *Pseudomonas*-derived rhamnolipids act as biosurfactants, altering surface hydrophobicity and preventing the accumulation of proteins and polysaccharides that form the conditioning film [128]. Similarly, *Halomonas* spp. secrete exopolysaccharides that compete with organic foulants for binding sites, effectively “masking” the surface. These interventions are critical for impeding the first step in the fouling cascade [129].

Bioactive molecules derived from marine microorganisms that exhibit anti-fouling activity are a promising resource as they are non-toxic or less toxic than the antifouling compounds currently in use. Recently, Rico-Virgen et al. [130] found that seven crude extracts produced from bacteria isolated from marine organisms exhibited high antifouling potential. Cultured epibiotic bacteria incorporated into a Phytagel™ matrix inhibited the settlement of 67 diatom taxa belonging to 45 genera involved in forming the conditioning film. The results showed that the greatest number of diatoms occurred on the negative control plates, indicating that marine bacterial extracts exhibit effective antifouling properties and could be a sustainable alternative to the chemicals currently in use. Chemical characterization revealed these active extracts contained saponins, terpenes, sterols, and proteins, likely responsible for their inhibitory effects. They also investigated a critical aspect of antifouling research: toxicity assessment. To this end, bioassays were performed on *Artemia franciscana* using various concentrations of bacterial extracts. The results showed that only one of the seven extracts tested exhibited toxic effects, while the others were considered suitable for potential future use in antifouling treatments.

Compounds extracted from *Bacillus licheniformis* MBR1 and *Vibrio alginolyticus* MBR4 isolated from soft coral and macroalgae, respectively, inhibited the growth of biofilm-forming bacteria, biofilm formation, and barnacle larval settlement [131], targeting the inhibition of fouling at the *microbial biofilm formation* step. The GC-MS analysis of the extract detected the presence of compounds such as tetrapentacontane, octadecanoic acid, 2,3-dihydroxypropyl ester, hexadecanoic acid, 2-hydroxy-1-(hydroxymethyl) ethyl ester, and 17-pentatriacontene. As a further recent result, a rarely occurring sponge-associated bacterium of the genus *Alcanivorax* was isolated from the sponge *Siphonochalina siphonella* and subjected to a metabolite extraction procedure. Metabolites were evaluated for antibacterial and antibiofilm activities against five different biofilm-forming bacteria isolated from the microfouling assemblage. The GC-MS profiles showed that antibiofilm active metabolites are mostly fatty acids and their derivatives, such as tetradecanoic acid, dodecanoic acid, and hexadecanol from the culture supernatant extract [132].

A study by Alemán-Vega et al. [133] has shown that biosurfactants produced by marine bacteria exhibit antibiofilm activity and consequently antifouling potential. This could provide an environmentally friendly alternative to traditional antifouling paints. Like bacterial extracts, biosurfactants are of particular interest in this field due to their low toxicity and biodegradability. The study reported that the marine bacterium *Bacillus niabensis* exhibited high emulsifying activity and low toxicity toward *Artemia franciscana*. These combined properties make it an ideal candidate for antifouling and antibiofilm applications. This study employed an innovative technique involving the incorporation of marine microbial extracts, specifically cell-free supernatants (CFS), into paint formulations. One of the most effective CFSs was that of Bacillus niabensis, which increased the efficiency of the paint by 30%.

Numerous antifouling strategies have been developed so far, including micro/nanostructured surfaces, natural antifoulants, bioinspired hydrogels, slippery liquid-infused porous surfaces, dynamic surfaces inspired by biology, and zwitterionic coatings [134]. However, research and experimentation on effective natural alternatives against biofouling and biofilm formation are now more urgent than ever, especially with a focus on environmental sustainability and reducing the ecotoxicological impact of conventional solutions.

Research in the development of antifouling biocides is increasingly adopting the tools and methodologies of pharmaceutical chemistry, aiming to achieve compounds with greater selectivity and specificity of action [135]. Despite significant advances in the discovery of new antibiofilm and antifouling agents, their translation into clinical applications remains limited by challenges such as unsatisfactory biocompatibility profiles and insufficient bioavailability.

#### 3.5.1. Elasnin

Elasnin is a natural compound featuring an α-pyrone core, produced by *Streptomyces mobaraensis*. Recent studies have identified its efficacy as an inhibitor of marine biofilm formation. The molecule exhibits selective action, demonstrating activity against Gram-positive bacteria (MBIC: 1.25–2.5 μg/mL), while showing no significant efficacy against Gram-negative bacteria such as *E. coli* and *P. aeruginosa*. Its primary mechanism of action consists of the destruction of the biofilm matrix without direct lethal effects on the bacterial cells, which are instead released into the culture medium [136,137].

Currently, research on elasnin as an anti-biofilm agent remains confined to the preclinical stage. However, its application potential is protected by intellectual property rights. The patent document (https://patents.google.com/patent/US12342819B2/en?q=(elasnin)&oq=elasnin accessed on 10 September 2025), identified by the numbers CN112425608B and US12342819B2, describes in detail elasnin-based compositions and methods for their use in inhibiting the formation and eradicating bacterial biofilms.

#### 3.5.2. Tambjamines

Tambjamines are yellow pigments isolated from *Pseudoalteromonas tunicata*, and they have biological activities such as antifouling (against *S. aureus*, *E. coli*), anti-microorganism, anti-invertebrate larva, antialgal spore, anti-protozoan, and antifungal activities, making them desirable targets for pharmaceutical agents [138]. The presence of pigmented metabolites like tambjamines in Pseudoalteromonas is particularly interesting because the genus is commonly associated with macroorganisms, such as algae, fish, sponges and tunicates [139]. Tambjamine pigment has not yet reached any clinical stage of development for human use. Its application is still firmly confined to pre-clinical research. The patent document (https://patents.google.com/patent/WO2016176450A1/en?q=(Tambjamines)&oq=Tambjamines accessed on 10 September 2025) describes the methodology for the synthesis of the compound.

## 4. Application of Marine Bacterial Bioactive Compounds

### 4.1. Pharmacological Treatments

Approximately 60% of all antibiotics produced by pharmaceutical companies have been obtained from cultures of *Actinobacteria* strains. Strains of the *Streptomyces* sp. alone have been the source of over half of all antibiotics used in pharmaceutical applications. Many studies are actively searching for new antibiotics. One notable example is anthramycin, an antibiotic isolated from *Streptomyces* sp. with strong inhibitory effects against *Bacillus anthracis*, the bacterium whose spores cause anthrax, a lethal disease in humans [37]. Although most antibiotic compounds exhibit antibacterial effects, some also show associated antitumor activity. Of particular interest is the *Salinispora* sp., which is associated with certain marine invertebrates and algae [140]. The first compound isolated from this bacterium, known as Salinosporamide A, has demonstrated strong cytotoxic effects against various cancer cell lines, including NCI-H226 lung cancer, SF-539 brain tumors, SK-MEL-28 melanoma, and MDA-MB-43 breast cancer [141]. Cyanobacteria are also worthy of particular interest, given that they produce extremely active peptides, including dolastatin, the most potent naturally synthesized compound in treating lymphomas and similar oncological diseases [142].

Bioactive metabolites produced by associated bacteria demonstrate robust antibacterial and antifungal activities, thereby protecting the sponge from pathogenic microorganisms. It is noteworthy that *Streptomyces* sp. and *Salinispora* sp. residing in sponges produce metabolites that are effective against pathogens such as *Candida albicans* [143] and *Staphylococcus aureus* [144].

Similarly, mollusks-associated bacteria, particularly *Streptomyces* sp. in cone snails, synthesize defensive compounds such as polyketide pyrones that protect the host from predation and environmental threats [145].

In fish, bioactive compounds derived from marine bacteria have shown efficacy in combating key aquaculture pathogens, particularly those of the genus *Vibrio*, such as *V. anguillarum* and *V. harveyi*, which are responsible for vibriosis outbreaks. For example, certain biosurfactants and antimicrobial peptides produced by marine *Bacillus* and *Pseudoalteromonas* strains have demonstrated anti-*Vibrio* activity by inhibiting biofilm formation and quorum sensing pathways [146]. These metabolites can disrupt bacterial communication and adhesion, thereby preventing colonization of gill and intestinal surfaces in fish. Such effects reduce infection incidence and mortality in aquaculture settings, potentially offering natural alternatives to traditional antibiotics and contributing to more sustainable fish farming practices. The study by Meisel et al. [147] examines how the behavior and physiology of a simple organism can be influenced by its microbial environment. Their findings suggest that secondary metabolites produced by microbes are involved in pathogen recognition and host survival. Specifically, the study proposes that the chemosensory detection of two secondary metabolites from *Pseudomonas aeruginosa* modulates a neuroendocrine signaling pathway that encourages avoidance behavior in the host organism, *C. elegans*. In particular, the metabolites phenazine-1-carboxamide and pyochelin activate the G-protein signaling pathway in the ASJ chemosensory neurons, which in turn triggers the expression of the neuromodulator DAF-7/TGF-β. DAF-7 subsequently activates a typical TGF-β signaling pathway in nearby interneurons, adjusting the host’s aerotaxis behavior and promoting avoidance of *P. aeruginosa*.

These findings highlight the broad ecological and biomedical importance of marine microbial metabolites. These metabolites not only protect aquatic organisms from infection but also influence host behavior and survival via intricate neuroendocrine signaling processes. Future research should explore these interactions further, focusing on the potential application of these metabolites as immunomodulatory or probiotic agents in sustainable aquaculture systems.

### 4.2. Industrial Applications

Bioactive compounds from marine bacteria are used in the production of enzymes, pigments, and other useful products for industrial applications, such as the production of bioplastics. These compounds are a commercially promising and sustainable alternative to those sourced from plants and animals. They provide better performance under industrial conditions, such as extreme temperature, pH, pressure, and salinity, even at lower concentrations [25]. The advanced use of bioplastics is still developing, especially in the fields of creating bioplastics that can resist food pathogens and in drug delivery systems [148].

In this context, polyhydroxybutyrate (PHB) shows promising applications in bioplastic production. PHB is a biodegradable polymer with physical and mechanical properties similar to conventional plastics, offering a sustainable alternative to petroleum-derived plastics. PHB is synthesized via the condensation of two acetyl-CoA molecules to acetoacetyl-CoA by the acetyl-CoA acetyltransferase, which is then reduced to (R)-3-hydroxybutyrate-CoA by the acetoacetyl-CoA reductase. This is followed by the polymerization of (R)-3-hydroxybutyrate-CoA to PHB by the PHB synthase. As microbial strains accumulate PHB as a survival mechanism, an ecosystem with extreme stress conditions, such as a marine environment, could contain superior PHB producers. For instance, marine bacteria, such as *Erythrobacter aquimaris*, can accumulate PHB in high-salt and nutrient-limited environments, enhancing production efficiency [149]. This ability positions marine-derived PHB as a viable bioplastic for environmental applications, particularly in industries like agriculture and medicine, where biodegradable materials are increasingly prioritized. Marine bacteria that have recently attracted attention for their ability to produce PHB are *Alteromonas lipolytica*, *Bacillus megaterium*, *Halomonas campaniensis*, *Pseudomonas* spp., *Aeromonas* spp., *Cupriavidus* spp. *Archaebacteria*, *Methylobacteria and Pseudomonas* [150].

An example of a microbial surface-active biopolymer that has reached commercial production is xanthan gum, a hydrocolloid produced by the plant-pathogenic bacterium *Xanthomonas campestris*. In addition to its interfacial properties, xanthan gum acts as a viscosity builder, making it a key ingredient in various healthcare products and food processing formulations [151]. Another surface-active biopolymer, which has been shown to exert excellent emulsifying properties and has reached commercial production is emulsan, produced by *Acinetobacter venetianus* RAG-1. Emulsan contains hydrophobic components, such as fatty acids, attached to its polysaccharide backbone, giving it amphiphilic properties. Among low-molecular-weight (LMW) surface-active agents, rhamnolipids and sophorolipids have also been commercialized for use in cleaning products [152]. 

### 4.3. Production of Cosmetic Products

A variety of compounds from marine bacteria, including polyketides, alkaloids, peptides, proteins, lipids, mycosporines, mycosporine-like amino acids, glycosides, isoprenoids, and hybrid molecules, have strong potential for use in producing cosmetic products. These compounds provide benefits such as sun protection, anti-aging effects, anti-microbial properties, antioxidant activity, and moisturizing effects [153]. It is well documented that prolonged exposure to UVA (320–400 nm) and UVB (280–320 nm) radiation can cause immediate and long-term effects on the skin and overall human health. A variety of marine organisms have evolved the capacity to shield themselves from deleterious UV radiation through the synthesis of UV-absorbing compounds, including scytonemin (a unique pigment produced by cyanobacteria), carotenoids, and melanin [154]. These compounds have the potential to serve as novel UV filters for sunscreens. In particular, photosynthetic organisms have been extensively investigated as a source of these compounds [152]. Marine carotenoids, known for their strong antioxidant and anti-inflammatory properties, can help protect the skin from harmful effects caused by UV radiation. To meet the rising demand for these beneficial compounds, recent advances in engineering have significantly boosted carotenoid production. Notably, certain marine heterotrophic bacteria, particularly from the genera *Paracoccus* and *Agrobacterium*, show promise as efficient sources of astaxanthin, a valuable carotenoid [155].

The defining characteristic of anti-aging formulations is the prevalence of moisturizing substances, which are vital for the maintenance of epidermal cellular functions. Among these substances, marine organisms produce several high-molecular-weight molecules, including polysaccharides, fatty acids (PUFA, sophorolipids, rhamnolipids and mannosyl-erythritol), and proteins like collagen. These are widely used in skin care due to their ability to soften and smooth the skin. These substances include, for instance, exopolysaccharides (EPS) and fatty acids. Particularly, EPS produced by the marine bacterium *Alteromonas macleodii* (HYD657) are commonly used in cosmetics. Similarly, a combination of EPS derived from *Pseudoalteromonas* strains, isolated from Antarctic waters and obtained through fermentation, has been demonstrated to stimulate collagen I production, thereby enhancing dermal structure [156]. Other anti-ageing products employ the use of EPS derived from marine microbes, including *Pseudoalteromonas antarctica*, *Halomonas eurihalina* and the deep-sea bacterium *Vibrio diabolicus* which produces a hexosaccharide (HE800) with structural similarities to hyaluronic acid [157].

### 4.4. Bioremediation

Bioremediation is the set of environmentally friendly strategies that employs the use of microorganisms, plants, or their enzymes to degrade, detoxify, or remove pollutants from contaminated environments, including soil, water, and air. The process utilizes the intrinsic microbial metabolic capabilities to facilitate the breakdown of harmful substances into less toxic or innocuous by-products, thereby rendering it an efficacious approach for the remediation of oil spills, heavy metals, pesticides, and other pollutants [158].

In this context, BSs play a pivotal role. In the case of hydrocarbons, for example, they increase the solubility of hydrophobic pollutants, making them more available for microbial degradation. Concerning heavy metals, however, BSs can promote mobilization by chelating metal ions, thereby rendering them more accessible for subsequent bioremediation processes [159]. Recent studies have focused on benthic filter-feeding organisms as potential sources for isolating biosurfactant-producing bacterial strains. These organisms, through their intense filtering activity, accumulate pollutants from the surrounding water, fostering the growth of bacterial communities capable of degrading or removing these contaminants. In this context, Rizzo et al. [160] reported biosurfactant-producing bacteria isolated from polychaetes (*Megalomma claparedei*, *Sabella spallanzanii*, and *Branchiomma luctuosum*), including previously unreported genera in the context of BSs such as *Cellulophaga*, *Cobetia*, *Cohaesibacter*, *Idiomarina*, *Pseudovibrio*, and *Thalassospira*. Notably, two strains of *Idiomarina*—*Idiomarina* sp. 185, isolated from Antarctic sediments, and *Idiomarina* sp. A19, isolated from a temperate environment (Lake Faro, Messina), have recently been recognized for their ability to produce biosurfactants at low and moderate temperatures, as well as in mineral media supplemented with biphenyl, a class of pollutants related to polychlorinated biphenyls (PCBs) [161]. These findings suggest the potential for applying these strains in the remediation of environments contaminated with polychlorinated biphenyls (PCBs).

EPS are also reported to have heavy metal chelation activity, a property that enhances their environmental and biotechnological relevance. Studies have explored the functions and applications of EPS in organisms from polar environments [92] and marine hydrothermal vents [99]. For example, the strain *Pseudoalteromonas* sp. MER144, isolated from Antarctic marine waters, is a prolific EPS producer, along with strains like *Winogradskyella* sp. CAL384 and CAL396, *Colwellia* sp. GW185, and *Shewanella* sp. CAL606, isolated from Antarctic sponges [92]. The EPS produced by these bacterial strains demonstrates key biotechnological properties, such as emulsifying activity, cryoprotection, and most notably, the ability to chelate heavy metals, making them promising agents for applications in bioremediation and environmental protection [94].

Despite recent advances in the study of biosurfactants, many remain undiscovered, and research strategies still require refinement. A deeper understanding is essential for characterizing biosynthetic mechanisms, defining chemical structures, and identifying novel molecular frameworks with potential biological activities and as-yet-unknown biotechnological applications [162,163].

### 4.5. Anti-Fouling Strategies

Marine bioactive compounds produced by *Pseudoalteromonas* sp. have been observed to exhibit notable effects across a variety of organisms and ecological interactions. For instance, a significant activity of this bacterial species is the inhibition of fouling organisms [128]. The use of compounds such as violacein and brominated compounds has been demonstrated to discourage the colonization of competitive microorganisms and prevent the attachment and growth of invertebrates, including barnacles and tube worms. These effects help to keep the surfaces of marine organisms clean, such as algae and sponges, which host *Pseudoalteromonas* sp. as symbionts. This anti-fouling capacity is also observed in the formation of biofilms, where these compounds limit bacterial succession, thereby reducing biofilm accumulation on both natural and artificial marine surfaces. Moreover, bioactive metabolites from *Pseudoalteromonas* sp. impact predator-prey interactions. Compounds such as pentabromopseudilin can be lethal to small eukaryotes, including protozoa and invertebrates that feed on *Pseudoalteromonas* sp. This reduces predation pressure and increases bacterial survival. The production of these bioactive substances not only contributes to competitive dominance within the microbial community but also serves to maintain ecological balance by regulating the populations of fouling and predator species [128].

Although some strains belonging to the class of marine *Actinobacteria* are known to produce both antibacterial and antiviral compounds, the study by Neu et al. [164] aimed to assess their toxic effects. Indeed, the impact of secondary metabolites produced by these organisms on *Artemia* sp. and *Caenorhabditis elegans* was assessed to identify the specific compounds responsible for the observed toxicity. One of the most toxic strains identified is *Pseudoalteromonas luteoviolacea* S4060, which demonstrated a rapid impact on *Artemia*, resulting in the death of all individuals within four hours. This toxic effect is attributed to pentabromopseudiline, an antibacterial compound that has been demonstrated to interfere with motor activity by inhibiting myosin ATPase. This inhibition results in paralysis, indicating a significant neuromuscular alteration.

A recent study by Ayuningrum et al. [165] revealed that marine bacteria associated with tunicates represent a source of antimicrobial compounds that exert a significant impact on the health and ecology of the host organism. These bioactive metabolites assist the tunicates in resisting pathogenic invasions, thereby maintaining their overall health and resilience in the marine environment. In addition to providing localized protection for the host, these compounds contribute to broader ecological dynamics by establishing a microenvironment on the surface of the tunicate that limits biofouling and the growth of potential pathogens.

Algae-associated bacteria have been shown to produce bioactive bacterial compounds that can function as algicides, inhibiting the growth of harmful algal blooms or as growth stimulants in controlled settings, indicating their role in both ecosystem regulation and aquaculture applications. In this context, compounds like NIG355 and Soforolipid, produced by *Streptomyces malaysiensis*, have been demonstrated to possess curative actions against the algae *Phaeocystis globosa* and *Aeromicrobium tamlense* [166].

## 5. Biodiscovery Pipeline: The Path for Concrete Application

While numerous studies in the scientific literature have focused on the discovery and characterization of bioactive molecules of microbial origin, this review aims to shift the focus toward the subsequent steps that follow the initial identification of a novel compound. These steps are essential for advancing the compound toward real-world applications or potential market introduction. In particular, when considering the use of such compounds in bioremediation, it becomes critical to evaluate their safety. Assessing the non-toxicity of newly discovered molecules is a fundamental requirement, and therefore cytotoxicity testing on model cell lines and organisms should be integrated as a standard component of the biodiscovery pipeline.

### 5.1. Methods

**Figure 2:**
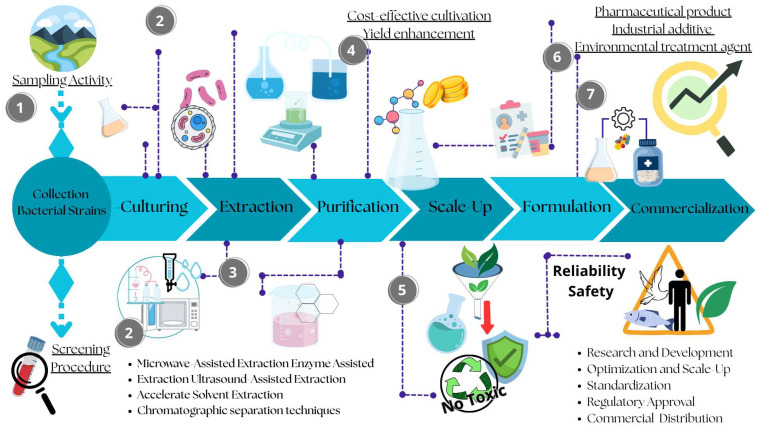
Representation of the biodiscovery pipeline steps: from culturing to formulation and commercialization.

Once the potential of marine bacteria as a source of bioactive molecules of biotechnological interest has been recognized, it is important to emphasize that it is not enough to only discover a bioactive molecule, as its specific activity must also be identified. Each discovered molecule must in fact be extracted by appropriate methods that preserve its structural and functional integrity, tested and finally screened. Its production under laboratory conditions must be optimized by following specific procedures for each category of molecule, especially for those to be used in the medical, pharmaceutical, nutraceutical or cosmeceutical fields [167]. Figure 2 shows a detailed map of the steps required in a biodiscovery pipeline, starting from the culturing of marine bacteria and describing the technologies for extracting the target molecules and the types of tests for screening them in model biological systems.

Search and extraction are scientific activities aimed at identifying, isolating and recovering molecules or bioactive compounds produced by bacteria or other organisms. They involve different steps and methods depending on the origin and derivation of the microorganisms, if they are of bacterial origin. Once the environment and origin of the target bacteria have been identified, a crucial first step is to obtain a collection of bacterial strains. In the case of the marine environment, collections can come from different sources, such as seabed sediments at different depths, water columns, and strains associated with benthic and nectonic organisms.

Traditional extraction techniques are well-established and long-used methods for extracting bioactive compounds. Some of these techniques include solvent extraction, one of the most common methods, in which bacterial cultures or supernatants obtained from them are treated with organic solvents (i.e., ethyl acetate, ethanol, acetone) to extract the bioactive compounds. In contrast, distillation is used to extract volatile compounds, in which the bacterial cultures or supernatants obtained from them are heated and then the steam is condensed and collected [168,169]. Although these traditional extraction techniques are widely used, they often have limitations, such as the use of large quantities of solvent or very long extraction times. Therefore, in recent years, new, more efficient and environmentally friendly extraction technologies have been developed to improve both the yield and purity of the extracts, including Microwave-Assisted Extraction [170], Enzyme Assisted Extraction (EAE) [171], Ultrasound-Assisted Extraction (UAE) [172], Accelerate Solvent Extraction (ASE) [173], Chromatographic separation techniques [174].

After extraction, the metabolite must be purified to remove any contaminants and obtain a purer form of the compound. Once this is achieved, the metabolite needs to be analyzed by biological and chemical screening to determine its chemical structure and bioactive properties [167]. Chemical screening consists of searching for new chemical structures without considering their biological activity. The chemical properties of isolated compounds, obtained by the previous methods, could be analyzed by means of optical techniques such as UV, mass spectrometry (MS) [175], and nuclear magnetic resonance (NMR) [176] or chromatography. For biological activity screening, much depends on the type of bioactive molecule being studied and the end application of interest. In the case of BSs or EPSs as an example, the main screening tests are aimed at evaluating the interfacial, emulsifying, and foaming activity through specific assays, i.e., surface tension measurement, stable emulsion production, oil spreading assay, and drop oil test. Conversely, in the case of compounds with potential applications in medical and pharmaceutical fields, the screening should include the investigation of antibacterial activity (i.e., plate diffusion agar method, minimum inhibitory concentration, overlay assay) or anti-proliferative activity against cancer and tumor cells. In the case of molecules with anti-biofilm activity, screening tests must be used to ascertain the inhibitory or destructive activity of microbial biofilms, while for the anti-fouling activity, the ability of the extract to intervene in the attachment and development processes of organisms involved in fouling must be explored.

To transition from laboratory research to commercial use, the compound must be produced on a larger scale through scale-Up and formulation steps. This requires bioprocess optimization, including cost-effective cultivation, yield enhancement, and purification. The compound is then formulated based on its intended application—whether as a pharmaceutical product, industrial additive, or environmental treatment agent.

Developing therapies based on these metabolites involves significant challenges. These include understanding their pleiotropic functions, determining optimal delivery methods and dosages, and addressing variability in individual responses. Additionally, inhibiting harmful metabolite production is hindered by limited tools to target microbial enzymes. Time-resolved and context-specific studies are essential to tailor effective interventions. Despite these hurdles, harnessing microbiome-derived metabolites offers a largely untapped potential to influence disease outcomes.

### 5.2. Approval

Before reaching the market, natural bioactive products must undergo safety assessments and regulatory approvals, depending on their application and geographic region. For pharmaceuticals, this includes preclinical and clinical trials, while industrial and environmental products must meet safety, toxicity, and environmental impact standards.

The path for market integration requires some steps in addition to the first previously treated in this text. The experimental procedures including the identification and characterization of marine bacterial strains and their bioactive compounds, supported by genomics, proteomics, and advanced screening methods represent the Research and Development phase, followed by the already mentioned Optimization and Scale-Up phase, aimed at optimizing bacterial growth conditions, enhancing metabolite yields, and developing cost-effective fermentation or bioprocessing systems for assess feasibility of large-scale production. To carry out these steps at least three other phases are necessary. The stability, safety and effectiveness of the active ingredients in the product should be ensured and demonstrated through the Product Formulation and Standardization, which involves formulating the product in a way that preserves its bioactivity and ensures consistent quality.

Regulatory Approval is then necessary for marine bacterial products before entering the market, and the regulations vary depending on the product type (e.g., food, pharmaceutical, cosmetic) and region but typically include toxicological assessments and environmental impact studies. The final step is the Commercial Partnerships and Distribution, which requires active collaboration with industry partners that is crucial for marketing, distribution, and scale. Licensing agreements, joint ventures, and tech-transfer initiatives help bring marine bacterial innovations to market.

As can be seen, a lot of effort has to be put in even after the discovery of a bioactive compound, and considering that the use of microorganisms greatly facilitates the process due to the rapid growth rates and the possibility of growing cells in the laboratory and enhancing the optimal production conditions, it is worth going beyond the initial process. In this respect, however, much remains to be done. In order to test the reliability and safety of a product, toxicity tests must be carried out to ensure that the product will not cause further damage if released into the environment or tested on a living organism.

## 6. Effects of Marine Bacterial Bioactive Compounds on Model Organisms

As pointed out in previous sections, research and discoveries in the field of marine biodiscovery are skewed towards studies aimed at isolating new compounds with potential medical or pharmaceutical applications. In fact, most of the products registered as inventions or patents fall into the category of medicines or antibiotics. In this area, the post-discovery process seems to be clearer and less controversial. In fact, in this context, to obtain final approval, a compound must undergo various stages of clinical trials before it can be applied and marketed. From this point of view, compounds that can be applied in other areas, such as environmental recovery, antifouling, and antibiofilm in the environment, remain under-investigated. While, as we have observed, marine compounds are generally much less widely recognized than terrestrial compounds in the medical and pharmaceutical fields, this is even more true in other areas of application. However, the use of natural compounds in other industrial fields also deserves attention. For example, if countless examples of biosurfactants or EPS with chelating action against heavy metals or hydrocarbon-degrading properties were to be used in strategies for the recovery of contaminated environments, it would be necessary to prove that they have no cytotoxic effects for direct use in the environment. In this context, testing on model organisms can be of great help in giving a real boost to the market entry of compounds with competitive and promising characteristics.

Discoveries made using model organisms often provide important insights that can be applied to understanding the biology of higher organisms. Model organisms are invaluable for acquiring fundamental biological knowledge, screening pharmacologically and biotechnologically relevant molecules, and conducting experiments that would be challenging or impractical to perform directly on more complex organisms [177]. Additionally, they exhibit a range of characteristics that replicate pathological conditions, thereby facilitating the investigation of novel drugs, the enhancement of therapeutic protocols, and the creation of medical devices for the treatment and management of human diseases. Model organisms may also be cell lines or isolated molecules (such as DNA, proteins, or enzymes of specific metabolic pathways) selected and simplified to be easier to study, manipulate, and analyze than more complex organisms or biological contexts. They are useful tools for acquiring basic biological knowledge, for screening molecules of pharmacological and biotechnological interest, or for conducting experiments that might be difficult or impractical to perform directly on more complex organisms [177,178,179].

Discoveries made using model organisms often provide important information that can be applied to understanding the biology of higher organisms. Moreover, model organisms have multiple aspects that mimic various pathological conditions and thus enable the study of new drugs, the optimization of therapeutic protocols, and the development of medical devices for the cure and treatment of human diseases. The model organism has an enormous use in biomedical research as it allows the in vivo study of toxicological tests, dose–response analyses, and the evaluation of side effects of bioactive molecules and drugs in the whole organism.

In this context, model organisms are a valuable resource in biomedical research, enabling in vivo studies for toxicological testing, dose–response analyses, and the evaluation of side effects of bioactive molecules and drugs on the entire organism [180]. The yeast *Saccharomyces cerevisiae*, the fruit fly *Drosophila melanogaster*, the nematode *Caenorhabditis elegans*, the ascidian *Ciona intestinalis*, the Mediterranean mussel *Mytilus galloprovincialis*, and the zebrafish *Danio rerio*, are among the most widely used model organisms in toxicological research [180]. In addition to their applications in toxicology, model organisms are also valuable in pharmacological research [181,182,183]. Moreover, cell lines or isolated molecules, such as DNA, proteins or enzymes of specific metabolic pathways, are regarded as highly efficacious models in experimental research due to their relative ease of study, manipulation, and analysis in comparison to more complex organisms or biological contexts. They are useful tools for acquiring fundamental biological knowledge, for screening molecules of pharmacological and biotechnological interest, or for conducting experiments that may be challenging or impractical to perform directly on more complex organisms [177].

Whereas for bioactive products with applications in the medical and pharmaceutical fields, tests on cell lines are more frequently carried out first to assess the effects of the extracts well, for bioactive compounds with potential application in the environmental field, it is essential to assess their non-toxicity. In this, the use of model species can be crucial, especially if the intended application is in the marine environment, as in the case of anti-fouling strategies. Hereinafter, we shortly report the main model organisms currently used to test the cytotoxicity of marine bacterial compounds.

### 6.1. Mytilus galloprovincialis

The use of *Mytilus galloprovincialis* as a model organism lies in many features, foremost among their easy cultivation both in the open sea and in tanks with very low costs. The developmental biology of this species also allows it to grow rapidly depending on the cultivation conditions, reaching considerable biomasses in a short time. Like all mussels, *M. galloprovincialis* is a filter-feeder, which exposes it to environmental stresses and the accumulation of significant amounts of various xenobiotics, such as pesticides, medicines, heavy metals, industrial compounds, antioxidants and antivirals found dissolved in seawater. Due to the hemolymph that transports these xenobiotics throughout the organism and the hemocytes that react to the foreign substance, this bivalve is capable of implementing various systemic responses such as changes in their shape, size, mortality rates, occurrence of histopathological lesions and even cell volume that can be easily studied. The main methods widely used to assess the toxic effects of other compounds are the trypan blue (TB) test and neutral red retention assay techniques (NRRA) [184].

The NRRA is a cytological test that allows the analysis of cell viability based on the ability of cells to retain Neutral Red dye within lysosomes, known as target organelles for the accumulation of aromatic hydrocarbons and organophosphates. Viable cells accumulate the dye in lysosomes with standardized retention times; this is due to the stability of the lysosomal membrane. Experimentally induced states of stress can alter the permeability status of this membrane and result in changes in dye retention times that can be related to stress response or pathological conditions [185].

The TB technique is also used to estimate cell viability. The plasma membrane of viable cells is impermeable to many dye molecules, including trypan blue, a naphthalenesulphonic acid; on cell death, the membrane loses its selective capacity and integrity, allowing the dye to penetrate the cytoplasm. Under a classic light microscope observation, viable cells will appear colorless, whereas dead cells will be deep blue. Deliberate treatments with cytotoxic molecules that affect cell viability can change the percentage of dead cells observable under the light microscope. However, this method is time-consuming and depends on the operator’s discretion. To overcome this, analysis by flow cytometry can be performed. It is known that TB binds to proteins and the TB-protein complex emits fluorescence that can be measured in a flow cytometer at a wavelength of 660 nanometers. The comparison of live vs. dead cells in this case is evaluated electronically and is therefore more reliable. However, this technique does not allow apoptotic cells to be distinguished from dead ones and can therefore lead to an underestimation of the mortality rate. For this reason, it is often accompanied by further analyses using other dyes such as propidium iodide, which binds specifically to nuclear DNA [186]. *M. galloprovincialis* is also an excellent model for assessing inflammatory states, levels of oxidant enzymes and antioxidant protections, mitochondrial function and membrane stability reflecting the impact of internal and external factors [187].

### 6.2. Ciona Intestinalis

Ascidians, or Tunicates, are marine animals considered a model for toxicity tests. Functional genetic studies have uncovered numerous genetic networks underlying neuronal specification and differentiation. One interesting part concerns the formation of the brain in ascidians, as it is sensitive to toxic agents. Modern techniques available for ascidians now allow the evaluation of numerous endpoints to test the specific mode of action of many compounds. The dynamic phenotypic changes of *Ciona* larvae during metamorphosis have been used to examine the effect of cytotoxic drugs on its development by quantifying six toxicity parameters [188]: degenerate tail size, ampulla length, body axis rotation, stomach size, heart rate and body size. As a result, mitochondrial respiration inhibitors, tubulin polymerization/depolymerization inhibitors or DNA/RNA synthesis inhibitors showed distinct toxicity profiles on these six parameters, but drugs with the same targets showed a similar toxicity profile in *Ciona*. *Ciona* is therefore suggested an effective animal model for drug toxicity profiling and for exploring the mechanisms of drugs with unknown targets. Numerous studies report exposure of *Ciona* to hundreds of drug types, suggesting that it is sufficiently sensitive to manifest drug-induced effects [189]. Different phenotypes of larvae are generally treated by monitoring toxicity parameters influenced by cytotoxic drugs.

### 6.3. Danio rerio

Among model organisms, the zebrafish (*Danio rerio*) is a prominent organism in recent biological research, used in research to study cancer, toxicology, drug discovery, and molecular genetics. This small fish is versatile for many research fields due to its easy maintenance, reproduction, and transparent body during early development. The zebrafish is a tropical freshwater fish inhabiting river, mainly the Ganges; it is a bony fish belonging to the family Cyprinidae. The identification of mutants is one of the most important strategies for studying various areas of biology. Zebrafish share many physiological and genetic similarities with humans, including the brain, digestive tract, musculature, vascularity, and innate immune system. In addition, the zebrafish genome shares many similarities with the human genome. Approximately 70% of disease-associated genes in humans have functional homologues in zebrafish [190]. The zebrafish is an ideal model for the study of cancer, being ably exploited for cancer treatment, transplantation of mammalian cancer cells, and transgenic regulation.

To the best of our knowledge, no studies on the test of cytotoxicity of marine bacterial extracts against model organisms were carried out, or information on this issue is scant. Despite the ecological importance of aquatic environments and the frequent release of bacterial metabolites into aquatic ecosystems, there is a paucity of recent studies analyzing the effects of these compounds on aquatic organisms. This knowledge gap hinders a comprehensive understanding of the potential impacts of bacterial bioactive compounds in marine ecosystems, or on the feasibility of their inclusion in diets or treatment plans, in the case of, for example, aquaculture activities. This section and Table 2 summarize the current state of the art on the effects of marine bacterial bioactive compounds on non-target organisms and the ecosystem.

**Table 2:** Effects of main marine bacterial bioactive compounds on non-target organisms and ecosystem.

Bioactive Compounds	Effects	Type and Chemical Structure	References
**Phloroglucinols**	Contributes to the biological control of plant disease	Phenolic benzenetriol; C_6_H_3_(OH)_3_	[191]
**EPS produced by *Bifidobacterium***	Regulator of interlukin-10 secretion, plays a vital role during inflammation	Protein–lipid complex	[192]
**Sebastenoic acid**	Acts as an antimicrobial agent against various pathogenic bacteria	Medium-chain fatty acid; C_10_H_18_O_4_	[192]
**Extracellular materials produced by *Pseudoalteromonas***	Inhibited the settlement and metamorphosis of the ubiquitous fouling invertebrate larvae, *Balanus amphitrite* and *Hydroides elegans*	glycolipids/protein–polysaccharide complexes	[193]
**256-tribromo-1-methylgramine**	Potent inhibitor against larval settlement of the barnacle *Amphi-balanus amphitrite*	Halogenated indole alkaloid; C_12_H_13_Br_3_N_2_	[193]
**Lichenicidin**	*Enterococcus faecalis*, *Salmonella typhimurium*, *Escherichia coli*, *Cronobacter sakazakii*	Consists of two peptides: Lchα (approximately C_130_H_191_N_35_O_39_S_5_) and Lchβ (roughly C_110_H_158_N_26_O_30_S_4_)	[194]
**Pentabromopseudilin**	Deadly to small eukaryotes, such as protozoa and invertebrates	Highly brominated bromophenol-bromopyrrole hybrid; C_10_H_4_Br_5_NO	[128]
**Soforolipid**	Possess curative actions against the algae *Phaeocystis globosa* and *Aeromicrobium tamlense*	Glycolipid biosurfactant; C_34_H_58_O_15_	[166]
**Dimethylsulfoniopropionate**	Aids in structuring the coral-associated bacterial community and enhancing the tolerance of corals to ROS and thermal stress	Organosulfur zwitterionic compound; C_5_H_10_O_2_S	[195]
**Thallusin**	Induces normal germination and morphogenesis of green macroalgae.	Sesquiterpenoid morphogen; C_25_H_31_NO_7_	[196]

## 7. New Approaches for Marine Biodiscovery

The traditional approach to discovering new bioactive compounds from bacteria is based on culture-dependent techniques. This starts with the isolation of bacteria, which can be difficult in the case of marine environments, followed by growth on specific culture media. Phenotypic screening to understand the requirements of the strains and the optimal conditions for producing metabolites is a crucial step in the process of extracting and purifying the compounds. Finally, in vitro and in vivo preclinical models are required to gain a deep understanding of their effects and the mechanism of action for the final application. However, this conventional method suffers from several limitations and needs to be integrated with other approaches. Firstly, it is well known that only a small percentage of marine bacteria can be cultivated in the laboratory. However, even these can be challenging to isolate in pure cultures and maintain under laboratory conditions.

In this context, the metagenomic approach is a culture-independent method that allows overcoming the problems related to cultivation conditions, and provides access to the genomes of uncultivable bacteria through direct sequencing and cloning techniques. This approach has advantages over traditional culture-based methods. Metagenomics is particularly more effective for studying bacteria from extreme environments, as their growth conditions are often difficult to replicate using conventional techniques due to their unique adaptations. Moreover, through a metagenomic approach, it is possible to detect specific genes or gene clusters within the genome involved in the biosynthesis pathways. As stated by Mohan et al. [54], it is thanks to metagenomics that genes encoding the polyketide synthase complex were detected within a bacterial symbiont called Candidatus *Endobugula sertula*, thus confirming its involvement in the production of the polyketide bryostatin 1, initially attributed to the bryozoan *Bugula neritina* [197]. Similar case is that referred to the Trabectedin EY-743, an anticancer drug isolated from a sea squirt *Ecteinascidia turbinate*, whose production was then attributed to the Gammaproteobacterium Candidatus *Endoecteinascida frumentensis*. In parallel, the improvement of the heterologous expression of pathways and the synthetic approach are emerging strategies of considerable interest.

The use of a host organism to regulate the expression of a gene or a gene fragment is recognized as a challenging perspective to obtain new useful products, and requires the development of new systems that can elicit activity from silent or cryptic gene clusters [198]. The advent of synthetic biology has expanded the possibilities for creating enhanced molecules with promising results [199,200]. The principle is based on modifying and refining existing natural products through genetic or chemical methods, therefore stimulating a strong emphasis in the integration of synthetic biology with marine biodiscovery. The development of synthetic compounds that imitate marine natural products, engineered organisms to produce natural compounds, and altered antimicrobial peptides represent some of the groundbreaking progress in this dynamic field of biodiscovery. Starting with a lead structure, is possible to undergo synthetic development to improve its activity, pharmacodynamics and pharmacokinetics, turning natural products into clinically effective drugs. Moreover, the coupling of molecular approach and synthetic biology offers additional possibility to boost the discovery in marine bioprospecting with the synthetic metagenomic, consisting in codon optimization of genes of interest followed by chemical synthesis, cloning and expression in an optimized heterologous host [201].

Finally, in the informatic era we are living in, the integration of artificial intelligence (AI) and machine learning technologies is transforming the field of marine bioprospecting, enabling the identification and prediction of novel bioactive compounds in a faster, cheaper and more targeted manner. Unlike traditional methods, which often involve culture, extraction and bioassay procedures, AI-based approaches can screen huge datasets derived from genomics, transcriptomics and metabolomics to uncover the biosynthetic potential of marine microorganisms, including non-culturable taxa [202]. Incorporating AI into marine bioprospecting enables accelerating the discovery pipeline, and this approach is particularly promising for exploring extreme or under-sampled marine ecosystems.

Among the most widely used tools are antiSMASH (Antibiotics and Secondary Metabolites Analysis Shell—Wageningen University & Research; Wageningen, The Netherlands) and PRISM (Prediction Informatics for Secondary Metabolomes—McMaster University, in Hamilton, ON, Canada) [203], which predict biosynthetic gene clusters of secondary metabolites from microbial genomes. These tools are increasingly being combined with deep learning platforms such as DeepBGC (Merck & Co, Rahway, NJ, USA) [204], which not only predicts the presence of secondary metabolites, but also classifies the chemical class and potential function of the predicted products. Another approach involves metabolomics-driven machine learning using software such as GNPS (Global Natural Products Social Molecular Networking—La Jolla, San Diego, CA, USA) and Spec2Vec (Wageningen, The Netherlands) [205], which pool MS/MS spectra to predict new metabolites or structural analogues based on existing compound libraries. Furthermore, AI models such as Chemprop (Massachusetts Institute of Technology; Cambridge, MA, USA) [206,207] and MoleculeNet (Stanford University, Stanford, CA, USA) [208] are able to predict the biological activity, toxicity, and physicochemical properties of a compound based on its molecular structure, helping in the initial validation phase and reducing the number of false positives.

## 8. Challenges and Future Perspectives

The marine environment is a very important source for scientific research on biotechnology, and specifically marine microorganisms. The present review focused on a selection of molecules of biotechnological interest produced by marine bacteria, and tried to provide an overview of the vastness of the biological processes underlying their production. Furthermore, it was clearly stated how much this research area is unexplored and how high the probability is that there are still producers not yet characterized and molecular structures that have not yet been defined. In this context, metagenomics represents a fundamental tool for overcoming the limitations imposed by traditional isolation and cultivation techniques. Through the direct analysis of DNA extracted from environmental samples, it is possible to access the genetic heritage of microorganisms that cannot be cultured in the laboratory, paving the way for the identification of new biosynthetic genes and potential bioactive molecules. Marine metagenomes thus constitute a strategic resource for the discovery of new metabolic pathways and the characterization of natural compounds with pharmacological, antimicrobial, anticancer, or enzymatic activity.

If, on the one hand, the marine environment provides us with sources of bioactive molecules, which can potentially be used in various fields of interest to man (including those more closely related to human health), on the other hand, it also provides us with the means to verify the concrete applicability of such molecules, through ideal model biological systems that allow their safety to be verified.

Another emerging aspect concerns the use of synthetic biology, which enables the redesign and optimization of metabolic pathways for the heterologous production of marine metabolites in genetically modified model organisms. This strategy helps overcome the limitations associated with low production yields under natural conditions and facilitates industrial scalability. Moreover, the combination of functional metagenomics and synthetic biology allows for the expression of “silent” biosynthetic genes that would otherwise remain unexpressed in the native organism. The new frontiers of blue biotechnologies call precisely for refining studies, making screening processes faster and less expensive, applying greener extraction techniques, thus also falling within the context of sustainability, which is very important in this era. Finally, it is important standardize and assess the molecule validation processes, making them faster but at the same time safe and resource-friendly. Challenges in the field include the complexity of compound isolation, variability in natural sources, production costs, and regulatory hurdles. However, advancements in synthetic biology, genetic engineering, environmental metagenomics, and sustainable extraction technologies are helping overcome these barriers. The future lies in the development of eco-friendly, high-performance bioactive products that meet the growing demand for sustainable solutions across multiple sectors.

## 9. Conclusions

In conclusion, the journey from the discovery of natural bioactive compounds to their commercialization involves a multidisciplinary approach that integrates biology, chemistry, engineering, and environmental science. These compounds hold immense promise for improving human health, industrial efficiency, and environmental sustainability. Despite their potential, marine bacterial products face several barriers to market integration, i.e., high research and production costs; regulatory complexity, especially for novel compounds; and the need for consumer education and industry awareness. The study of the literature and the search for specific patents or commercialized compounds highlighted a significant gap between the different categories of compounds. Those with medical or pharmaceutical applications are more prevalent in post-discovery pathways, probably because clinical trials are more clearly defined and established. Other categories of marine bacterial products, which are used in other fields, are mostly confined to the discovery phase. However, in some cases, these compounds also require direct use in the environment and should therefore be evaluated for toxicity to allow their use. Differently, in the case of compounds that may be useful for commercialization in the industrial field, cost/benefit assessments would be required during the discovery phase in order to encourage companies. Moreover, another interesting finding is that the isolated bacterial species are mostly affiliated with actinomycetes. This suggests the need to explore new sources to isolate new producers and/or compounds.

However, these challenges are being addressed through advancements in marine biotechnology, synthetic biology, and public–private partnerships. As sustainability becomes a major market driver, the demand for natural, eco-friendly, and marine-sourced solutions is expected to grow rapidly. A small step that could help speed up the process, however, is the standardization and inclusion of toxicity tests on model organisms, even for bioactive compounds of marine bacterial origin. This would make it possible to provide a product of proven efficacy, not only with optimal conditions and yields, but also with a first attempt of safety and reliability. Targeted testing on aquatic model organisms could be crucial for the implementation of bioremediation and anti-fouling strategies, ensuring that introduction into the sea does not affect other living organisms.

## Data Availability

No new data were created or analyzed in this study. Data sharing is not applicable to this review.

## References

[1] Ahmadniaye Motlagh H., Horie Y., Rashid H., Banaee M., Multisanti C.R., Faggio C. (2023). Unveiling the effects of fennel (*Foeniculum vulgare*) seed essential oil as a diet supplement on the biochemical parameters and reproductive function in female common carps (*Cyprinus carpio*). Water.

[2] Impellitteri F., Multisanti C.R., Riolo K., Zicarelli G., Porretti M., Cafeo G., Faggio C. (2025). Bergamot (*Citrus bergamia*): A potential new nutraceutical against cellular and physiological alterations induced by emerging contaminants in sentinel organisms. Antioxidants.

[3] Jindal R., Sharma R., Kaur P., Kaur S., Multisanti C.R., Faggio C. (2024). Mitigation of haemato-genotoxic and stress response effects in *Cyprinus carpio* via silymarin dietary supplementation following deltamethrin exposure. Heliyon.

[4] Rashidian G., Boldaji J.T., Rainis S., Prokić M.D., Faggio C. (2021). Oregano (*Origanum vulgare*) extract enhances zebrafish (*Danio rerio*) growth performance, serum and mucus innate immune responses and resistance against *Aeromonas hydrophila* challenge. Animals.

[5] Multisanti C.R., Riolo K., Impellitteri F., Zicarelli G., Vazzana I., Cafeo G., Giannetto A. (2025). Bergamot (*Citrus bergamia*) as a potential anti-stress agent: Counteracting cellular and physiological changes by sodium lauryl sulphate in *Mytilus galloprovincialis*. Environ. Pollut..

[6] Giri S.S., Sen S.S., Jun J.W., Sukumaran V., Park S.C. (2017). Role of *Bacillus licheniformis* VS16-derived biosurfactant in mediating immune responses in Carp Rohu and its application to the food industry. Front. Microbiol..

[7] Pastaki N.J., Abdollahpour H., Karimzadeh M., Zamani H., Multisanti C.R., Faggio C. (2023). Physiological and immunological impact of methanolic lavender extract on female goldfish (*Carassius auratus*). Aquac. Rep..

[8] Van Doan H., Prakash P., Hoseinifar S.H., Ringø E., El-Haroun E., Faggio C., Dawood M.A. (2023). Marine-derived products as functional feed additives in aquaculture: A review. Aquac. Rep..

[9] Vijayaram S., Razafindralambo H., Sun Y.Z., Piccione G., Multisanti C.R., Faggio C. (2024). Synergistic interaction of nanoparticles and probiotic delivery: A review. J. Fish. Dis..

[10] Xian Y., Zhang J., Bian Z., Zhou H., Zhang Z., Lin Z., Xu H. (2020). Bioactive natural compounds against human coronaviruses: A review and perspective. Acta Pharm. Sin. B.

[11] Jimoh A.A., Lin J. (2019). Biosurfactant: A new frontier for greener technology and environmental sustainability. Ecotoxicol. Environ. Saf..

[12] Gill B.S., Qiu F. (2020). Technologies for extraction and production of bioactive compounds. Biotechnological Production of Bioactive Compounds.

[13] Rizzo C., Lo Giudice A. (2018). Marine Invertebrates: Underexplored Sources of Bacteria Producing Biologically Active Molecules. Diversity.

[14] Andryukov B., Mikhailov V., Besednova N. (2019). The biotechnological potential of secondary metabolites from marine bacteria. J. Mar. Sci. Eng..

[15] Srinivasan R., Kannappan A., Gandhi A.D., Kannan R.R. (2021). Marine microbes as a valuable source for bioactive compounds. Front. Microbiol..

[16] Venter J.C. (2003). Environmental genome shotgun sequencing of the Sargasso Sea. Science.

[17] Newman D.J., Hill T. (2006). New drugs from marine microbes: The tide is turning. J. Ind. Microbiol. Biotechnol..

[18] Venturella G., Ferraro V., Cirlincione F., Gargano M.L. (2021). Medicinal mushrooms: Bioactive compounds, use, and clinical trials. Int. J. Mol. Sci..

[19] Demain A.L., Zhang L. (2005). Natural products and drug discovery. Natural Products: Drug Discovery and Therapeutic Medicine.

[20] Haefner B. (2003). Drugs from the deep: Marine natural products as drug candidates. Drug Discov. Today.

[21] Al-Dhabi N.A., Ghilan A.K.M., Esmail G.A., Arasu M.V., Duraipandiyan V., Ponmurugan K. (2019). Bioactivity assessment of the Saudi Arabian Marine *Streptomyces* sp. Al-Dhabi-90, metabolic profiling and its in vitro inhibitory property against multidrug resistant and extended-spectrum beta-lactamase clinical bacterial pathogens. J. Infect. Public Health.

[22] Andryukov B.G., Zaporozhets T.S., Besednova N.N. (2018). Promising strategies for finding new means of fighting with infectious diseases. Antibiot. Khimioter..

[23] Böhringer N., Fisch K.M., Schillo D., Bara R., Hertzer C., Grein F., Schäberle T.F. (2017). Antimicrobial potential of bacteria associated with marine sea slugs from North Sulawesi, Indonesia. Front. Microbiol..

[24] Rizzo M.G., Briglia M., Zammuto V., Morganti D., Faggio C., Impellitteri F., Graziano A.C.E. (2025). Innovation in osteogenesis activation: Role of marine-derived materials in bone regeneration. Curr. Issues Mol. Biol..

[25] Banat I.M., Franzetti A., Gandolfi I., Bestetti G., Martinotti M.G., Fracchia L., Marchant R. (2010). Microbial biosurfactants production, applications and future potential. Appl. Microbiol. Biotechnol..

[26] Glaser K.B., Mayer A. (2009). A renaissance in marine pharmacology: From preclinical curiosity to clinical reality. Biochem. Pharmacol..

[27] Rizzo C., Poli A., Di Donato P., Nicolaus B., Lo Giudice A. (2022). Biosurfactant-producing bacteria from extreme marine environments: The case of *Idiomarina* spp.. Mar. Drugs.

[28] Lo Giudice A., Gugliandolo C. (2019). A special issue on microorganisms from extreme environments in Memory of Luigi Michaud (1974–2014). Diversity.

[29] Giordano D. (2020). Bioactive molecules from extreme environments. Mar. Drugs.

[30] Srinivasan R., Kannappan A., Shi C., Lin X. (2021). Marine bacterial secondary metabolites: A treasure house for structurally unique and effective antimicrobial compounds. Mar. Drugs.

[31] Raghukumar S., Raghukumar S. (2017). Extreme marine environments. Fungi in Coastal and Oceanic Marine Ecosystems: Marine Fungi.

[32] World Health Organization (2019). Monitoring and Evaluation of the Global Action Plan on Antimicrobial Resistance: Framework and Recommended Indicators.

[33] Chokshi A., Sifri Z., Cennimo D., Horng H. (2019). Global contributors to antibiotic resistance. J. Glob. Infect. Dis..

[34] Wibowo J.T., Bayu A., Aryati W.D., Fernandes C., Yanuar A., Kijjoa A., Putra M.Y. (2023). Secondary metabolites from marine-derived bacteria with antibiotic and antibiofilm activities against drug-resistant pathogens. Mar. Drugs.

[35] Blunt J.W., Carroll A.R., Copp B.R., Davis R.A., Keyzers R.A., Prinsep M.R. (2018). Marine natural products. Nat. Prod. Rep..

[36] Haque N., Parveen S., Tang T., Wei J., Huang Z. (2022). Marine Natural Products in Clinical Use. Mar. Drugs.

[37] Stonik V.A., Makarieva T.N., Shubina L.K. (2020). Antibiotics from marine bacteria. Biochemics.

[38] Tacconelli E., Carrara E., Savoldi A., Harbarth S., Mendelson M., Monnet D.L., Zorzet A. (2018). Discovery, research, and development of new antibiotics: The WHO priority list of antibiotic-resistant bacteria and tuberculosis. Lancet Infect. Dis..

[39] ul Hassan S.S., Anjum K., Abbas S.Q., Akhter N., Shagufta B.I., Shah S.A.A., Tasneem U. (2017). Emerging biopharmaceuticals from marine actinobacteria. Environ. Toxicol. Pharmacol..

[40] Jang K.H., Nam S.J., Locke J.B., Kauffman M.C.A., Beatty M.D.S., Paul M.L.A., Fenical W. (2013). Anthracimycin, a potent anthrax antibiotic from a marine-derived actinomycete. Angew. Chem..

[41] Zhang B., Wang K.B., Wang W., Bi S.F., Mei Y.N., Deng X.Z. (2018). Discovery, biosynthesis, and heterologous production of streptoseomycin, an anti-microaerophilic bacteria macrodilactone. Org. Lett..

[42] Karuppiah V., Sun W., Li Z. (2016). Natural products of actinobacteria derived from marine organisms. Stud. Nat. Prod. Chem..

[43] Swain S.S., Paidesetty S.K., Padhy R.N., Singh P.K. (2017). Computational approach for locating effective cyanobacterial compounds against Mycobacterium tuberculosis. Indian. J. Pharmac Edu Res..

[44] Shin D., Byun W.S., Moon K., Kwon Y., Bae M., Um S., Lee S.K., Oh D.C. (2018). Coculture of Marine *Streptomyces* sp. with *Bacillus* sp. produces a new piperazic acid-bearing cyclic peptide. Front. Chem..

[45] Moncheva P., Tishkov S., Dimitrova N., Chipeva V., Antonova-Nikolova S., Bogatzevska N. (2002). Characteristics of soil actinomycetes from Antarctica. J. Cult. Collect..

[46] O’Brien A., Sharp R., Russell N.J., Roller S. (2004). Antarctic bacteria inhibit growth of food-borne microorganisms at low temperatures. FEMS Microbiol. Ecol..

[47] Nedialkova D., Naidenova M. (2004). Screening the antimicrobial activity of Actinomycetes strains isolated from Antarctica. J. Cult. Collect..

[48] Biondi N., Tredici M.R., Taton A., Wilmotte A., Hodgson D., Losi D., Marinelli F. (2008). Cyanobacteria from benthic mats of Antarctic lakes as a source of new bioactivities. J. Appl. Microbiol..

[49] Lo Giudice A.L., Bruni V., Michaud L. (2007). Characterization of Antarctic psychrotrophic bacteria with antibacterial activities against terrestrial microorganisms. J. Basic Microbiol..

[50] Shekh R.M., Singh P., Singh S.M., Roy U. (2010). Antifungal activity of Arctic and Antarctic bacteria isolates. Polar Biol..

[51] Dong N., Di Z., Yu Y., Yuan M., Zhang X., Li H. (2013). Extracellular enzyme activity and antimicrobial activity of culturable bacteria isolated from soil of Grove Mountains, East Antarctica. Acta Microbiol. Sin..

[52] Encheva M., Zaharieva N., Kenarova A., Chipev N., Chipeva V., Hristova P., Ivanova I., Moncheva P. (2013). Abundance and activity of soil actinomycetes from Livingston Island, Antarctica. Bulg. J. Agric. Sci..

[53] Papaleo M.C., Fondi M., Maida I., Perrin E., Giudice A.L., Michaud L., Mangano S., Bartolucci G., Romoli R., Fani R. (2012). Sponge-associated microbial Antarctic communities exhibiting antimicrobial activity against *Burkholderia cepacia* complex bacteria. Biotechnol. Adv..

[54] Mohan S., Ajay Krishna M.S., Chandramouli M., Keri R.S., Patil S.A., Ningaiah S., Somappa S.B. (2023). Antibacterial natural products from microbial and fungal sources: A decade of advances. Mol. Divers..

[55] Banerjee A., Mohammed Breig S.J., Gómez A., Sánchez-Arévalo I., González-Faune P., Sarkar S., Cabrera-Barjas G. (2022). Optimization and characterization of a novel exopolysaccharide from *Bacillus haynesii* CamB6 for food applications. Biomolecules.

[56] Bister B., Bischoff D., Ströbele M., Riedlinger J., Reicke A., Wolter F. (2004). Abyssomicin C-A polycyclic antibiotic from a marine Verrucosispora strain as an inhibitor of the p-aminobenzoic acid/tetrahydrofolate biosynthesis pathway. Angew. Chem. Int. Ed..

[57] Keller S., Nicholson G., Drahl C., Sorensen E., Fiedler H.-P., Süssmuth R.D. (2007). Abyssomicins G and H and atrop-abyssomicin C from the marine Verrucosispora strain AB-18-032. J. Antibiot..

[58] Wang Q., Song F., Xiao X., Huang P., Li L., Monte A. (2013). *Abyssomicins* from the South China sea deep-sea sediment *Verrucosispora* sp.: Natural thioether michael addition adducts as antitubercular prodrugs. Angew. Chem. Int. Ed..

[59] León B., Navarro G., Dickey B.J., Stepan G., Tsai A., Jones G.S. (2015). Abyssomicin 2 reactivates latent HIV-1 by a PKC-and HDAC-independent mechanism. Org. Lett..

[60] Zhang D., Xu J., Qin Q., An F., Wang S., Li L., Lin H. (2022). *Marinacarboline glucuronide*, a new member of β-carboline alkaloids from sponge-derived actinomycete *Actinoalloteichus cyanogriseus* LHW52806. J. Antibiot..

[61] Waksman S.A., Woodruff H.B. (1940). Bacteriostatic and Bactericidal Substances Produced by a Soil Actinomyces. Soc. Exp. Biol. Med..

[62] Xianqin S., Jiang X., Sun J., Zhang C., Zhang Y., Lu L., Ju J. (2017). Study on the secondary metabolites of antibacterial activity of the actinomycete *Stremptmeces costaricanus* SCSIOZS0073. Nat. Prod. Res. Dev..

[63] Zhou L., Chang Y., Yang S. (2024). Antibacterial p-terphenyl and α-pyrone derivates isolated from the marine-derived actinomycete Nocardiopsis sp. HDN154086. J. Antibiot..

[64] Zhou X., Tang L., Fang W., Lee K., Liu Y., Luo X. (2019). Two Piericidin Glucosides and Application Thereof to Anti-Kidney Cancer Medicines.

[65] Janso J.E., Haltli B.A., Eustáquio A.S., Kulowski K., Waldman A.J., Zha L., Nakamura H., Bernan V.S., He H., Carter G.T. (2014). Discovery of the lomaiviticin biosynthetic gene cluster in *Salinispora pacifica*. Tetrahedron.

[66] Kersten R.D., Lane A.L., Nett M., Richter T.K.S., Duggan B.M., Dorrestein P.C., Moore B.S. (2013). Bioactivity-guided genome mining reveals the lomaiviticin biosynthetic gene cluster in *Salinispora tropica*. ChemBioChem.

[67] He H., Ding W.D., Bernan V.S., Richardson A.D., Ireland C.M., Greenstein M., Ellestad G.A., Carter G.T. (2001). Lomaiviticins A and B, potent antitumor antibiotics from *Micromonospora lomaivitiensis*. J. Am. Chem. Soc..

[68] Xue M., Herzon S.B. (2016). Mechanism of nucleophilic activation of (-)-lomaiviticin A. J. Am. Chem. Soc..

[69] Potts B.C., Albitar M.X., Anderson K.C., Baritaki S., Berkers C., Bonavida B., Chandra J., Chauhan D., Cusack J.C., Fenical W. (2011). Marizomib, a proteasome inhibitor for all seasons: Preclinical profile and a framework for clinical trials. Curr. Cancer Drug Targets.

[70] Piskorz W.M., Kretowski R., Cechowska-Pasko M. (2024). Marizomib *(Salinosporamide A)* Promotes Apoptosis in A375 and G361 Melanoma Cancer Cells. Mar. Drugs.

[71] Richardson P.G., Todd M., Zimmerman C.C., Hofmeister M., Talpaz A.A., Chanan-Khan J.L., Kaufman J.P., Laubach D., Chauhan A.J., Jakubowiak S. (2016). Phase 1 study of marizomib in relapsed or relapsed and refractory multiple myeloma: NPI-0052-101 Part 1. Blood.

[72] Feling R.H., Buchanan G.O., Mincer T.J., Kauffman C.A., Jensen P.R., Fenical W. (2003). Salinosporamide A: A highly cytotoxic proteasome inhibitor from a novel microbial source, a marine bacterium of the new genus *Salinospora*. Angew. Chem. Int. Ed..

[73] Zhou X., Tang L., Fang W., Lee K., Liu Y. (2019). Two Piericidin Glucosides and Application Thereof to Anti-Kidney Cancer Medicines.

[74] Zheng L., Xu Y., Lin X., Ning R.N., Zhen W., Zhang T. (2021). Marine Brevibacillus Brevis Anti-Tumor Active Polypeptide and Medicine and Application Thereof.

[75] McBrien K.D., Berry R.L., Lowe S.E., Neddermann K.M., Bursuker I., Huang S., Klohr S.E., Leet J.E. (1995). Rakicidins, new cytotoxic lipopeptides from *Micromonospora* sp. fermentation, isolation and characterization. J. Antibiot..

[76] Igarashi Y., Shimasaki R., Miyanaga S., Oku N., Onaka H., Sakurai H., Saiki I., Kitani S., Nihira T., Wimonsiravude W. (2010). Rakicidin D, an inhibitor of tumor cell invasion from marine-derived *Streptomyces* sp.. J. Antibiot..

[77] Oku N., Matoba S., Yamazaki Y.M., Shimasaki R., Miyanaga S., Igarashi Y. (2014). Complete stereochemistry and preliminary structure-activity relationship of rakicidin A, a hypoxia-selective cytotoxin from *Micromonospora* sp.. J. Nat. Prod..

[78] Kitani S., Ueguchi T., Igarashi Y., Leetanasaksakul K., Thamchaipenet A., Nihira T. (2018). Rakicidin F, a new antibacterial cyclic depsipeptide from a marine sponge-derived *Streptomyces* sp.. J. Antibiot..

[79] Chen L., Zhao W., Jiang H., Kim C., Fung R.L., Hong J., Gang L. (2021). Natural Rakicidins Compound Rakicidin I and Extraction Method Thereof.

[80] Chen L., Zhao W., Jiang H., Kim C., Fung R.L., Hong J., Gang L. (2021). Natural Rakicidins Compound Namely Rakicidin H and Extraction Method Thereof.

[81] Chen L., Lin F., Jiang H., Zhao V., Kim C., Honglei J., Gang L. (2021). Natural Rakicidins Compound Rakicidin B1-2 and Fermentation and Extraction Method Thereof.

[82] Romero F., Espliego F., Pérez Baz J., García de Quesada T., Grávalos D., De La Calle F. (1997). Thiocoraline, a new depsipeptide with antitumor activity produced by a marine *Micromonospora* I. Taxonomy, fermentation, isolation, and biological activities. J. Antibiot..

[83] Wyche T.P., Hou Y., Braun D., Cohen H.C., Xiong M.P., Bugni T.S. (2011). First natural analogs of the cytotoxic thiodepsipeptide thiocoraline A from a marine *Verrucosispora* sp.. J. Org. Chem..

[84] Ameen F., AlNadhari S., Al-Homaidan A.A. (2021). Marine microorganisms as an untapped source of bioactive compounds. Saudi J. Biol. Sci..

[85] Dawson S., Malkinson J.P., Paumier D., Searcey M. (2007). Bisintercalator natural products with potential therapeutic applications: Isolation, structure determination, synthetic and biological studies. Nat. Prod. Rep..

[86] Harunari E., Komaki H., Igarashi Y. (2016). Biosynthetic origin of anthracimycin: A tricyclic macrolide from *Streptomyces* sp.. J. Antibiot..

[87] Corral P., Amoozegar M.A., Ventosa A. (2019). Halophiles and Their Biomolecules: Recent Advances and Future Applications in Biomedicine. Mar. Drugs.

[88] Santos J.D., Vitorino I., Reyes F., Vicente F., Lage O.M. (2020). From ocean to medicine: Pharmaceutical applications of metabolites from marine bacteria. Antibiotics.

[89] Barzkar N., Sukhikh S., Babich O. (2024). Study of marine microorganism metabolites: New resources for bioactive natural products. Front. Microbiol..

[90] Jianhua J., Liu M., Sun Z., Li Q., Ma J. (2020). Didemethylated Actinomycin Derivative and Application Thereof in Preparation of Drugs for Resisting Drug-Resistant Bacteria Infection.

[91] Sheng G.P., Yu H.Q., Li X.Y. (2010). Extracellular polymeric substances (EPS) of microbial aggregates in biological wastewater treatment systems: A review. Biotechnol. Adv..

[92] Caruso C., Rizzo C., Mangano S., Poli A., Di Donato P., Finore I., Nicolaus B., Di Marco G., Michaud L., Giudice A.L. (2017). Production and Biotechnological Potential of Extracellular Polymeric Substances from Sponge-Associated Antarctic Bacteria. Appl. Environ. Microbiol..

[93] Kalinovskaya N.I., Romanenko L.A., Kalinovsky A.I., Ermakova S.P., Dmitrenok P.S., Afiyatullov S.S. (2017). The antitumor antibiotics complex of aureolic acids from the marine sediment-associated strain of Streptomyces sp. KMM 9048. Nat. Prod. Commun..

[94] Caruso C., Rizzo C., Mangano S., Poli A., Di Donato P., Nicolaus B., Lo Giudice A. (2019). Isolation, characterization and optimization of EPSs produced by a cold-adapted *Marinobacter* isolate from Antarctic seawater. Antarct. Sci..

[95] Gui C., Zhang S., Zhu X., Ding W., Huang H., Gu Y.C., Ju J. (2017). *Antimicrobial spirotetronate* metabolites from marine-derived *Micromonospora harpali* SCSIO GJ089. J. Nat. Prod..

[96] Caruso C., Rizzo C., Mangano S., Poli A., Di Donato P., Nicolaus B., Lo Giudice A. (2018). Extracellular polymeric substances with metal adsorption capacity produced by *Pseudoalteromonas* sp. MER144 from Antarctic seawater. Environ. Sci. Pollut. Res..

[97] Lo Giudice A., Poli A., Finore I., Rizzo C. (2020). Peculiarities of extracellular polymeric substances produced by Antarctic bacteria and their possible applications. Appl. Microbiol. Biotechnol..

[98] Jeewon R., Aullybux A.A., Puchooa D., Nazurally N., Alrefaei A.F., Zhang Y. (2023). Marine Microbial Polysaccharides: An Untapped Resource for Biotechnological Applications. Mar. Drugs.

[99] Lelchat F., Cozien J., Le Costaouec T., Brandilly C., Schmitt S., Baudoux A., Colliec-Jouault S., Boisset C. (2015). Exopolysaccharide biosynthesis and biodegradation by a marine hydrothermal *Alteromonas* sp. Strain. Appl. Microb. Biotechnol..

[100] Senni K., Gueniche F., Changotade S., Septier D., Sinquin C., Ratiskol J., Lutomski D., Godeau G., Guezennec J., Colliec-Jouault S. (2013). Unusual glycosaminoglycans from a deep sea hydrothermal bacterium improve fibrillar collagen structuring and fibroblast activities in engineered connective tissues. Mar. Drugs..

[101] Floris R., Rizzo C., Lo Giudice A.L. (2018). Biosurfactants from marine microorganisms. Metabolomics—New Insights into Biology and Medicine.

[102] Franzetti A., Tamburini E., Banat I.M. (2010). Applications of biological surface-active compounds in remediation technologies. Biosurfactants.

[103] Banat I.M., Satpute S.K., Cameotra S.S., Patil R., Nyayanit N.V. (2014). Cost effective technologies and renewable substrates for biosurfactants’ production. Front. Microbiol..

[104] Eras-Muñoz E., Farré A., Sánchez A., Font X., Gea T. (2022). Microbial biosurfactants: A review of recent environmental applications. Bioengineered.

[105] Marchant R., Banat I.M. (2012). Microbial biosurfactants: Challenges and opportunities for future exploitation. Trends Biotechnol..

[106] Silva N.M.P., Rufino R.D., Luna J.M., Santos V.A., Sarubbo L.A. (2013). Screening of *Pseudomonas* species for biosurfactant production using low-cost substrates. Biocatal. Agric. Biotechnol..

[107] Voulgaridou G.P., Mantso T., Anestopoulos I., Klavaris A., Katzastra C., Kiousi D.E., Pappa A. (2021). Toxicity profiling of biosurfactants produced by novel marine bacterial strains. Int. J. Mol. Sci..

[108] Cheng T.H., Ismail N., Kamaruding N., Saidin J., Danish-Daniel M.J.B.R. (2020). Industrial enzymes-producing marine bacteria from marine resources. Biotechnol. Rep..

[109] Dumorné K., Córdova D.C., Astorga-Eló M., Renganathan P. (2017). Extremozymes: A Potential Source for Industrial Applications. J. Microbiol. Biotechnol..

[110] Al-Agamy M.H., Alhuzani M.R., Kelany M.S., Hamed M.M. (2021). Production and Partial Characterization of α-Amylase Enzyme from Marine Actinomycetes. Biomed. Res. Int..

[111] Raddadi N., Cherif A., Daffonchio D., Neifar M., Fava F. (2015). Biotechnological applications of extremophiles, extremozymes and extremolytes. Appl. Microbiol. Biotechnol..

[112] Ghattavi S., Ahmad H. (2023). Marine enzymes: Classification and application in various industries. Int. J. Biol. Macromol..

[113] Thebti W., Riahi Y., Gharsalli R., Belhadj O. (2016). Screening and characterization of thermo-active enzymes of biotechnological interest produced by thermophilic *Bacillus* isolated from hot springs in Tunisia. Acta Biochim. Pol..

[114] Choudhary K., Mankar M.K., Sahay S. (2022). Extremophilic enzymes: Catalytic features and industrial applications. Extremophilic Fungi: Ecology, Physiology and Applications.

[115] Mirete S., Morgante V., González-Pastor J.E. (2017). Acidophiles: Diversity and mechanisms of adaptation to acidic environments. Adaption of Microbial Life to Environmental Extremes: Novel Research Results and Application.

[116] de Lourdes Moreno M., Pérez D., García M.T., Mellado E. (2013). Halophilic bacteria as a source of novel hydrolytic enzymes. Life.

[117] Daoud L., Ali M.B. (2020). Halophilic microorganisms: Interesting group of extremophiles with important applications in biotechnology and environment. Physiological and Biotechnological Aspects of Extremophiles.

[118] Sharma A., Kawarabayasi Y., Satyanarayana T. (2012). Acidophilic bacteria and archaea: Acid stable biocatalysts and their potential applications. Extremophiles.

[119] Kang H.K., Seo C.H., Park Y. (2015). Marine peptides and their anti-infective activities. Mar. Drugs.

[120] Shields R.C., Mokhtar N., Ford M., Hall M.J., Burgess J.G., El Badawey M.R., Jakubovics N.S. (2013). Efficacy of a marine bacterial nuclease against biofilm forming microorganisms isolated from chronic rhinosinusitis. PLoS ONE.

[121] Wang S., Zhao Y., Breslawec A.P., Liang T., Deng Z., Kuperman L.L., Yu Q. (2023). Strategy to combat biofilms: A focus on biofilm dispersal enzymes. npj Biofilms Microbiomes.

[122] Baslé A., Hewitt L., Koh A., Lamb H.K., Thompson P., Burgess J.G., Lewis R.J. (2018). Crystal structure of NucB, a biofilm-degrading endonuclease. Nucleic Acids Res..

[123] Canellas A.L.B., Dias G.R., Lopes I.R., Freitas-Silva J., Dobson A.D., Laport M.S., de Oliveira B.F.R. (2025). Marine microbial enzymes as potential antibiofilm agents: Expanding the arsenal of bioactive agents targeting biofilm-forming microorganisms. Crit. Rev. Microbiol..

[124] Blanco-Cabra N., Paetzold B., Ferrar T., Mazzolini R., Torrents E., Serrano L., LLuch-Senar M. (2020). Characterization of different alginate lyases for dissolving Pseudomonas aeruginosa biofilms. Sci. Rep..

[125] Yun S., Huang J., Zhang M., Wang X., Wang X., Zhou Y. (2024). Preliminary identification and semi-quantitative characterization of a multi-faceted high-stability alginate lyase from marine microbe *Seonamhaeicola algicola* with anti-biofilm effect on Pseudomonas aeruginosa. Enzym. Microb. Technol..

[126] Anjung M.U.K., Nursyam H., Prihanto A.A., Chasanah E. (2025). Application of marine bacterial alginate lyases in wastewater treatment, biofilm removal and green technology. Glob. J. Environ. Sci. Manag..

[127] Inoue A., Nishiyama R., Ojima T. (2016). The alginate lyases FlAlyA, FlAlyB, FlAlyC, and FlAlex from Flavobacterium sp. UMI-01 have distinct roles in the complete degradation of alginate. Algal Res..

[128] Bowman J.P. (2007). Bioactive compound synthetic capacity and ecological significance of marine bacterial genus *Pseudoalteromonas*. Mar. Drugs.

[129] Flemming H.C., Wingender J. (2010). The biofilm matrix. Nat. Rev. S Microbiol..

[130] Rico-Virgen E.G., Ortiz-Aguirre I., Hellio C., Rangel-Dávalos C., Escobedo-Fregoso A., López-Fuerte F.O., Aguila-Ramírez R.N. (2024). Antifouling activity of marine bacterial extracts: A non-toxic alternative as biocide. Rev. Biol. Mar. Oceanogr..

[131] Tunkal R.I., El-Sheekh M.M., Soliman A.M., Soliman M.S., Abu El-Kheir W.A., Abdel-Raouf N. (2023). Antifouling activity of bacterial extracts associated with soft coral and macroalgae from the Red Sea. Oceanol. Hydrobiol. Stud..

[132] Singh R., Dubey A.K. (2020). Isolation and characterization of a new endophytic actinobacterium *Streptomyces californicus* strain ADR1 as a promising source of anti-bacterial, anti-biofilm and antioxidant metabolites. Microorganisms.

[133] Alemán-Vega M., Sánchez-Lozano I., Hernández-Guerrero C.J., Hellio C., Quintana E.T. (2020). Exploring antifouling activity of biosurfactants producing marine bacteria isolated from Gulf of California. Int. J. Mol. Sci..

[134] Jin H., Tian L., Bing W., Zhao J., Ren L. (2022). Bioinspired marine antifouling coatings: Status, prospects, and future. Prog. Mater. Sci..

[135] Cahill P.L., Moodie L.W., Hertzer C., Pinori E., Pavia H., Hellio C., Svenson J. (2024). Creating new antifoulants using the tools and tactics of medicinal chemistry. Acc. Chem. Res..

[136] Long L., Sulaiman J.E., Xiao Y., Cheng A., Wang R., Malit J.J., Qian P.Y. (2022). Mode of action of elasnin as biofilm formation eradicator of methicillin-resistant *Staphylococcus aureus*. Front. Microbiol..

[137] Long L., Wang R., Chiang H.Y., Ding W., Li Y.-X., Chen F., Qian P.-Y. (2021). Discovery of Antibiofilm Activity of Elasnin against Marine Biofilms and Its Application in the Marine Antifouling Coatings. Mar. Drugs.

[138] Setiyono E., Adhiwibawa M.A.S., Prabowo M.R., Brotosudarmo T.H. (2021). Characterization of Tambjamines Pigment from Marine Bacterium *Pseudoalteromonas* sp. PM2 Indigenous from Alor Island, Indonesia. Indones. J. Nat. Pigment..

[139] Sakai-Kawada F.E., Ip C.G., Hagiwara K.A., Awaya J.D. (2019). Biosynthesis and bioactivity of prodiginine analogs in marine bacteria, *Pseudoalteromonas*: A mini review. Front. Microbiol..

[140] Kim H., Kim S., Kim M., Lee C., Yang I., Nam S.J. (2020). Bioactive natural products from the genus *Salinospora*: A review. Arch. Pharm. Res..

[141] Wang J., Shi Y., Jiang D. (2021). β-Lactone derivatives and their anticancer activities: A short review. Curr. Top. Med. Chem..

[142] Qamar H., Hussain K., Soni A., Khan A., Hussain T., Chénais B. (2021). *Cyanobacteria* as natural therapeutics and pharmaceutical potential: Role in antitumor activity and as nanovectors. Molecules.

[143] Öner Ö., Ekiz G., Hameş E., Demir V., Gübe Ö., Özkaya F., Yokeş M., Uzel A., Bedir E. (2014). Cultivable sponge-associated actinobacteria from coastal area of Eastern Mediterranean Sea. Adv. Microbiol..

[144] Asolkar R.N., Schroeder D., Heckmann R., Lang S., Wagner-Doebler I., Laatsch H. (2004). Helquinoline, a new tetrahydroquinoline antibiotic from *Janibacter limosus* Hel 1. J. Antibiot..

[145] Lin Z., Flores M., Forteza I., Henriksen N.M., Concepcion G.P., Rosenberg G., Schmidt E.W. (2012). Totopotensamides, polyketide–cyclic peptide hybrids from a mollusk-associated bacterium *Streptomyces* sp.. J. Nat. Prod..

[146] Wu W., Hu C.Q., Tao B.H. (2001). Studies on marine bacterial metabolites with antibacterial activity. J. Trop. Oceanogr..

[147] Meisel J.D., Panda O., Mahanti P., Schroeder F.C., Kim D.H. (2014). Chemosensation of bacterial secondary metabolites modulates neuroendocrine signaling and behavior of *C. elegans*. Cell.

[148] Kalia V.C., Ray S., Patel S.K.S., Singh M., Singh G.P. (2019). The dawn of novel biotechnological applications of polyhydroxyalkanoates. Biotechnological Applications of Polyhydroxyalkanoates.

[149] Mostafa Y.S., Alrumman S.A., Otaif K.A., Alamri S.A., Mostafa M.S., Sahlabji T. (2020). Production and characterization of bioplastic by polyhydroxybutyrate-accumulating *Erythrobacter aquimaris* isolated from mangrove rhizosphere. Molecules.

[150] Możejko-Ciesielska J., Kiewisz R. (2016). Bacterial polyhydroxyalkanoates: Still fabulous?. Microbiol. Res..

[151] Garti N., Leser M.E. (1999). Natural hydrocolloids as food emulsifiers. Design and Selection of Performance Surfactants.

[152] Johansson I., Kjellin M. (2010). Surfactants from Renewable Resources.

[153] Imhoff J.F., Labes A., Wiese J. (2011). Bio-mining the microbial treasures of the ocean: New natural products. Biotechnol. Adv..

[154] Gao Q., Garcia-Pichel F. (2011). Microbial ultraviolet sunscreens. Nat. Rev. Microbiol..

[155] Rastogi R.P., Richa, Kumar A., Tyagi M.B., Sinha R.P. (2010). Molecular mechanisms of ultraviolet radiation-induced DNA damage and repair. J. Nucleic Acids.

[156] Suen Y.L., Tang H., Huang J., Chen F. (2014). Enhanced production of fatty acids and astaxanthin in *Aurantiochytrium* sp. by the expression of *Vitreoscilla* hemoglobin. J. Agric. Food Chem..

[157] Courtois A., Berthou C., Guézennec J., Boisset C., Bordron A. (2014). Exopolysaccharides isolated from hydrothermal vent bacteria can modulate the complement system. PLoS ONE.

[158] Martins A., Vieira H., Gaspar H., Santos S. (2014). Marketed marine natural products in the pharmaceutical and cosmeceutical industries: Tips for success. Mar. Drugs.

[159] Aliko V., Multisanti C.R., Turani B., Faggio C. (2022). Get rid of marine pollution: Bioremediation an innovative, attractive, and successful cleaning strategy. Sustainability.

[160] Rizzo C., Michaud L., Graziano M., De Domenico E., Syldatk C., Hausmann R., Lo Giudice A. (2015). Biosurfactant activity, heavy metal tolerance and characterization of *Joostella* strain A8 from the Mediterranean polychaete *Megalomma claparedei* (Gravier, 1906). Ecotoxicology.

[161] Rizzo C., Michaud L., Hörmann B., Gerçe B., Syldatk C., Hausmann R., Lo Giudice A. (2013). Bacteria associated with sabellids (*Polychaeta*: *Annelida*) as a novel source of surface active compounds. Mar. Pollut. Bull..

[162] Rizzo C., Papale M., Lo Giudice A. (2022). *Idiomarina* sp. isolates from cold and temperate environments as biosurfactant producers. J. Mar. Sci. Eng..

[163] Zykwinska A., Marchand L., Bonnetot S., Sinquin C., Colliec-Jouault S., Delbarre-Ladrat C. (2019). Deep-sea hydrothermal vent bacteria as a source of glycosaminoglycan-mimetic exopolysaccharides. Molecules.

[164] Neu A.K., Månsson M., Gram L., Prol-García M.J. (2014). Toxicity of bioactive and probiotic marine bacteria and their secondary metabolites in *Artemia* sp. and *Caenorhabditis elegans* as eukaryotic model organisms. Appl. Environ. Microbiol..

[165] Ayuningrum D., Liu Y., Riyanti, Sibero M.T., Kristiana R., Asagabaldan M.A., Schaeberle T.F. (2019). Tunicate-associated bacteria show a great potential for the discovery of antimicrobial compounds. PLoS ONE.

[166] Sun X.X., Choi J.K., Kim E.K. (2004). A preliminary study on the mechanism of harmful algal bloom mitigation by use of sophorolipid treatment. J. Exp. Mar. Biol. Ecol..

[167] Li X., Zhao H., Chen X. (2021). Screening of marine bioactive antimicrobial compounds for plant pathogens. Mar. Drugs.

[168] Valença C.A.S., Hollanda L.M., Jain S., Caramão E.B. (2022). Chromatographic Profiles of Ethyl Acetate Extracts Produced by *Bacillus* sp. Collected from the Mangroves in the Brazilian Northeast. J. Braz. Chem. Soc..

[169] Baümler E.R., Carrín M.E., Carelli A.A. (2016). Extraction of sunflower oil using ethanol as solvent. J. Food Eng..

[170] Raynie D.E. (2006). Modern extraction techniques. Anal. Chem..

[171] Sanjeewa K.A., Herath K.H.I.N.M., Kim Y.S., Jeon Y.J., Kim S.K. (2023). Enzyme-assisted extraction of bioactive compounds from seaweeds and microalgae. TrAC Trends Anal. Chem..

[172] Lavilla I., Bendicho C. (2017). Fundamentals of ultrasound-assisted extraction. Water Extraction of Bioactive Compounds.

[173] Luthria D., Vinjamoori D., Noel K., Ezzell J. (2019). Accelerated solvent extraction. Oil Extraction and Analysis.

[174] Kumari V.C., Patil S.M., Ramu R., Shirahatti P.S., Kumar N., Sowmya B.P., Patrick-Iwuanyanwu K.C. (2022). Chromatographic techniques: Types, principles, and applications. Analytical Techniques in Biosciences.

[175] De Hoffmann E. (2000). Mass spectrometry. Kirk-Othmer Encyclopedia of Chemical Technology.

[176] Hore P.J. (2015). Nuclear Magnetic Resonance.

[177] Belma P., Dina F., Emina A., Nermina Ž., Fahir B. (2019). Animal models in modern biomedical research. Eur. J. Pharm. Med. Res..

[178] Tagkalidou N., Multisanti C.R., Bleda M.J., Bedrossiantz J., Prats E., Faggio C., Raldúa D. (2024). Analyzing the effects of age, time of day, and experiment on the basal locomotor activity and light-off visual motor response assays in zebrafish larvae. Toxics.

[179] Stevanović M., Tagkalidou N., Multisanti C.R., Pujol S., Aljabasini O., Prats E., Faggio C., Raldúa D. (2025). Zebra_K, a kinematic analysis automated platform for assessing sensitivity, habituation and prepulse inhibition of the acoustic startle response in adult zebrafish. Sci. Total Environ..

[180] Mukherjee P., Roy S., Ghosh D., Nandi S.K. (2022). Role of animal models in biomedical research: A review. Lab. Anim. Res..

[181] Impellitteri F., Riolo K., Multisanti C.R., Zicarelli G., Piccione G., Faggio C., Giannetto A. (2024). Evaluating quaternium-15 effects on *Mytilus galloprovincialis*: New insights on physiological and cellular responses. Sci. Total Environ..

[182] Multisanti C.R., Riolo K., Impellitteri F., Chebbi I., Faggio C., Giannetto A. (2023). Short-term *in vitro* exposure of *Pinctada imbricata*’s haemocytes to quaternium-15: Exploring physiological and cellular responses. Environ. Toxicol. Pharmacol..

[183] Multisanti C.R., Zicarelli G., Caferro A., Filice M., Faggio C., Vazzana I., Impellitteri F. (2024). From personal care to coastal concerns: Investigating polyethylene glycol impact on mussel’s antioxidant, physiological, and cellular responses. Antioxidants.

[184] Multisanti C.R., Impellitteri F., Cannatà G., Cotugno A., Perugini M., Piccione G., Faggio C., Rizzo M.G. (2025). Discovering the effects of octylisothiazolinone: Analysis of physiological changes in the Mediterranean mussel (*Mytilus galloprovincialis*). Ecotoxicol. Environ. Saf..

[185] Multisanti C.R., Impellitteri F., Zicarelli G., Perugini M., Iovine M.A., Filice M.C., Faggio C. (2025). Toxicological assessment of 2-methylisothiazol-3 (2H)-one on physiological and antioxidant parameters in *Mytilus galloprovincialis*. Environ. Pollut..

[186] Avelar-Freitas B.A., Almeida V.G., Pinto M.C.X., Mourão F.A.G., Massensini A.R., Martins-Filho O.A., Brito-Melo G.E.A. (2014). Trypan blue exclusion assay by flow cytometry. Braz. J. Med. Biol. Res..

[187] Murgarella M., Puiu D., Novoa B., Figueras A., Posada D., Canchaya C. (2016). A first insight into the genome of the filter-feeder mussel *Mytilus galloprovincialis*. PLoS ONE.

[188] Liu L., Xiang J., Dong B., Natarajan P., Yu K., Cai N. (2006). *Ciona intestinalis* as an emerging model organism: Its regeneration under controlled conditions and methodology for egg dechorionation. J. Zhejiang Univ. Sci. B.

[189] Mizotani Y., Itoh S., Hotta K., Tashiro E., Oka K., Imoto M. (2015). Evaluation of drug toxicity profiles based on the phenotypes of ascidian *Ciona intestinalis*. Biochem. Biophys. Res. Commun..

[190] Khan F.R., Alhewairini S. (2018). Zebrafish (*Danio rerio*) as a model organism. Curr. Trends Cancer Manag..

[191] Keswani C., Singh H.B., García-Estrada C., Caradus J., He Y.W., Mezaache-Aichour S., Sansinenea E. (2020). Antimicrobial secondary metabolites from agriculturally important bacteria as next-generation pesticides. Appl. Microbiol. Biotechnol..

[192] Bhattarai H.D., Ganti V.S., Paudel B., Lee Y.K., Lee H.K., Hong Y.K., Shin H.W. (2007). Isolation of antifouling compounds from the marine bacterium, *Shewanella oneidensis* SCH0402. World J. Microbiol. Biotechnol..

[193] Xin X., Huang G., Zhou X., Sun W., Jin C., Jiang W., Zhao S. (2017). Potential antifouling compounds with antidiatom adhesion activities from the sponge-associated bacteria, *Bacillus pumilus*. J. Adhes. Sci. Technol..

[194] Rather I.A., Galope R., Bajpai V.K., Lim J., Paek W.K., Park Y.H. (2017). Diversity of marine bacteria and their bacteriocins: Applications in aquaculture. Rev. Fish. Sci. Aquacult..

[195] Wang J., Curson A.R., Zhou S., Carrión O., Liu J., Vieira A.R., Todd J.D. (2024). Alternative dimethylsulfoniopropionate biosynthesis enzymes in diverse and abundant microorganisms. Nat. Microbiol..

[196] Matsuo Y., Imagawa H., Nishizawa M., Shizuri Y. (2005). Isolation of an algal morphogenesis inducer from a marine bacterium. Science.

[197] Davidson S.K., Haygood M.G. (1999). Identification of sibling species of the bryozoan *Bugula neritina* that produce different anticancer bryostatins and harbor distinct strains of the bacterial symbiont “Candidatus *Endobugula sertula*”. Biol. Bull..

[198] Reen F.J., Gutiérrez-Barranquero J.A., Dobson A.D., Adams C., O'Gara F. (2015). Emerging concepts promising new horizons for marine biodiscovery and synthetic biology. Mar. Drugs..

[199] Rocha-Martin J., Harrington C., Dobson A.D., O’Gara F. (2014). Emerging strategies and integrated systems microbiology technologies for biodiscovery of marine bioactive compounds. Mar. Drugs..

[200] Paddon C.J., Keasling J.D. (2014). Semi-synthetic artemisinin: A model for the use of synthetic biology in pharmaceutical development. Nat. Rev. Microbiol..

[201] Iqbal H.A., Feng Z., Brady S.F. (2012). Biocatalysts and small molecule products from metagenomic studies. Curr. Opin. Chem. Biol..

[202] Gupta S., Kumar D. (2024). AI-Driven Bioprospecting of Microbial Resources. Genomic Intelligence.

[203] Blin K., Shaw S., Kautsar S.A., Medema M.H., Weber T. (2020). The antiSMASH database version 3: Increased taxonomic coverage and new query features for modular enzymes. Nucleic Acids Res..

[204] Hannigan G.D., Prihoda D., Palicka A., Soukup J., Klempir O., Rampula L., Bitton D.A. (2021). A deep learning genome-mining strategy for biosynthetic gene cluster prediction. Nucleic Acids Res..

[205] Basnet B.B., Zhou Z.Y., Wei B., Wang H. (2025). Advances in AI-based strategies and tools to facilitate natural product and drug development. Crit. Rev. Biotechnol..

[206] Swanson K., Liu G., Catacutan D.B., Arnold A., Zou J., Stokes J.M. (2024). Generative AI for designing and validating easily synthesizable and structurally novel antibiotics. Nat. Mach. Intell..

[207] Zhang H., Jiang S., Sun H., Li Y., Yao Z. (2025). Exploration of Novel Antimicrobial Agents against Foodborne Pathogens via a Deep Learning Approach. J. Agric. Food Chem..

[208] Olayo-Alarcon R., Amstalden M.K., Zannoni A., Bajramovic M., Sharma C.M., Brochado A.R., Müller C.L. (2025). Pre-trained molecular representations enable antimicrobial discovery. Nat. Commun..

